# Microenvironmental stiffness directs microtubule perturbation in chondrocyte mitosis via ILK-refilinB/Smad3 axis

**DOI:** 10.1038/s41413-025-00491-4

**Published:** 2026-01-30

**Authors:** Mengmeng Duan, Chenchen Zhou, Guanyue Su, Chunhe Zhang, Jie Ren, Qingjia Chi, Xiaojing Liu, Li Yang, Haiqing Bai, Yang Claire Zeng, Seongmin Kim, Yunhao Zhai, Crystal Yuri Oh, Adam Yongxin Ye, Yuting Chen, Longlong Si, Xiaoheng Liu, Jing Xie

**Affiliations:** 1 State Key Laboratory of Oral Diseases, National Clinical Research Center for Oral Diseases, West China Hospital of Stomatology, Sichuan University, Chengdu, China; 2 Institute of Biomedical Engineering, West China School of Basic Medical Sciences & Forensic Medicine, Sichuan University, Chengdu, China; 3 Department of Engineering Structure and Mechanics, School of Science, Wuhan University of Technology, Wuhan, China; 4 Laboratory of Cardiovascular Diseases, Regenerative Medicine Research Center, West China Hospital, Sichuan University, Chengdu, China; 5 111 project Laboratory of Biomechanics and Tissue Repair, Bioengineering College, Chongqing University, Chongqing, China; 6 Wyss Institute for Biologically Inspired Engineering at Harvard University, Boston, MA USA; 7 Department of Cancer Biology, Dana Farber Cancer Institute Biological Chemistry and Molecular Pharmacology, Harvard Medical School, Boston, MA USA; 8 Department of Genetics, Harvard Medical School, Boston, MA USA; 9 Program in Cellular and Molecular Medicine, Boston Children’s Hospital, Boston, MA USA; 10 The Howard Hughes Medical Institute, Boston Children’s Hospital, Boston, MA USA; 11 CAS Key Laboratory of Quantitative Engineering Biology, Shenzhen Institute of Synthetic Biology, Shenzhen Institute of Advanced Technology, Chinese Academy of Sciences, Shenzhen, China

**Keywords:** Bone quality and biomechanics, Pathogenesis

## Abstract

Cells actively sense and transduce microenvironmental mechanical inputs into chemical signals via cytoskeletal rearrangements. During these mechanosensation and mechanotransduction processes, the role of the actin cytoskeleton is well-understood, whereas the role of the tubulin cytoskeleton remains largely elusive. Here, we report the dynamic changes in microtubules in response to microenvironmental stiffness during chondrocyte mitosis. Mechanical stiffness was found to be coupled with microtubule generation, directing microtubule dynamics in mitotic chondrocytes. Refilin B was found to be a key regulator of microtubule assembly in chondrocytes in response to mechanical stiffness. It was found to play its role in microtubule formation via the p-Smad3 signaling pathway. Additionally, integrin-linked kinase (ILK), triggered by mechanical stiffness, was found to play an indispensable role in the process of microtubule dynamics mediated by refilin B. Our data emphasizes stiffness-mediated dynamic changes in the microtubules of chondrocytes in a quiescent state (G0) and at anaphase, which improves our understanding of the mechanical regulation of microtubule assembly during the chondrocyte cell cycle and provides insights into microenvironment mechanics during tissue maintenance, wound healing, and disease occurrence.

## Introduction

Cells are constantly exposed to physical forces such as mechanical strain, fluid shear stress, extracellular matrix (ECM) mechanics, and cell-intrinsic traction forces in their microenvironment.^1–3^ These mechanical forces affect many biological processes, including morphogenesis, tissue maintenance, wound healing, and disease occurrence.^4,5^ Cells actively sense external mechanical changes by pulling and pushing on the ECM through subcellular components including focal adhesion plaques (FAs), contractile stress fibers (SFs), and microtubules.^6^ FAs are distributed in discrete regions of the cell and provide locations for mechanical attachment to the ECM.^7^ This attachment is mediated by transmembrane proteins, including nearly 200 known proteins such as integrins, vinculin, talin, tensin, zyxin, paxillin, and focal adhesion kinase.^8^ The formation and stability of FAs depend on the strength of extracellular mechanical forces, and FAs influence the generation and distribution of SFs—a characteristic type of actin filament that generates internal mechanical forces through actomyosin interactions.^9^ In addition to SFs formed by the self-assembly of actin monomers, the actin meshwork generated by the polymerization of actin monomers at the cell boundary can also push the membrane forward, generate cell protrusions, and promote cell migration.^10^ This entire process of FA- and SF-mediated cellular mechanosensation and mechanotransduction triggered by ECM mechanical forces is widely accepted. However, the dynamic changes in microtubules induced by microenvironmental mechanics during the mitotic cell cycle remain poorly understood.

Microtubules are one of the three major types of cytoskeletal filaments, with an outer diameter of ~25 nm.^11^ They are hollow cylindrical structures with mechanical rigidity, determining their involvement in many cellular activities such as cell morphological remodeling, intracellular signaling, metabolism, organelle organization, and molecular transport.^12^ Importantly, microtubules are essential for mitotic and meiotic spindles, which ensure proper cell division,^13^ and axonemes, which provide the central molecular dynamics for cilia and flagella.^14,15^ Microtubules, composed of α- and β-tubulin heterodimers (~50 kD each), are highly dynamic, as they can elongate or shorten through the addition or removal of tubulin subunits at their ends.^16^ While contributing to internal cellular traction forces, microtubules do not function independently but act through binding to actin filaments, intermediate filaments, and protein cytolinkers via a diverse profile of microtubule-associated proteins, which determine internal cytoskeletal stiffness and mechanotransduction.^17^ Microtubules can regulate cell and tissue morphogenesis either individually or by binding to actin microfilaments. For example, epithelial morphogenesis and cell polarity require the participation of microtubules, in addition to Rho-family GTPase activity.^18,19^ During brain development, microtubules play an essential role in neuronal morphogenesis by providing physical support in shaping precise neural processes.^20,21^ Microtubules are also involved in supporting tenocyte projections, thereby protecting the integrity of the tendon extracellular matrix.^22^ Regarding cartilage, recent reports have focused on the role of microtubules in the formation of primary cilia, which sense biomechanical and biophysical cues in the microenvironmental niche and transmit numerous signals to mediate cartilage growth, homeostasis, differentiation and disease occurrence.^23,24^

Articular cartilage is a specialized connective tissue that mainly consists of type II collagen and proteoglycans.^25,26^ It bears and redistributes the tensile, compressive, and shear forces imposed by body weight and various physical activity, acting as a shock absorber and load distributor at the load-bearing interfaces of joints.^27,28^ The biomechanical and tribological performance of articular cartilage is attributed to its ECM structure and zonal heterogeneity.^29^ Chondrocytes are a unique cell type in cartilage. These cells sense various forms of mechanical stimulation and respond by secreting matrix components to facilitate normal cartilage homeostasis. When the external mechanical load exceeds the maximum load that chondrocytes can bear, they undergo pathological changes and even apoptosis, which weaken the secretion of proteoglycans, alter the collagen components of the ECM, depolymerize procollagen bundles, destroy the procollagen network of the ECM, and initiate the development of osteoarthritis (OA).^30,31^ In addition, the increased matrix stiffness due to local fibrosis, enhanced type I collagen production, and osteophyte formation as a result of accelerated abnormal ossification are hallmarks of OA progression.^32^ The stiffness of the cartilage matrix in patients with OA can increase by approximately sixfold compared to that in normal patients (~190.5 kPa vs. ~34.86 kPa), and cartilage matrix stiffness increases even further with increasing OA disease severity (~383.9 kPa in severely damaged areas vs. ~ 62.98 kPa in relatively normal areas).^33^ Although these studies indicate that stiffened ECM can mediate FA and SF generation in chondrocytes, the mechanism by which it regulates microtubules remains largely unknown. In this study, we fabricated silicon-based elastomer polydimethylsiloxane (PDMS) substrates with different stiffnesses (soft substrate, ~46 kPa and stiff substrate, ~450 kPa) to characterize microtubule changes in chondrocyte mitosis and determine the associated biological pathways and potential mediators. Our study may help elucidate the mechanical regulation of microtubules in the cell mitotic cycle and identify novel mechanopathways that contribute to OA pathogenesis.

## Results

### Microtubule formation is coupled to mechanical stiffness

**Fig. 1:**
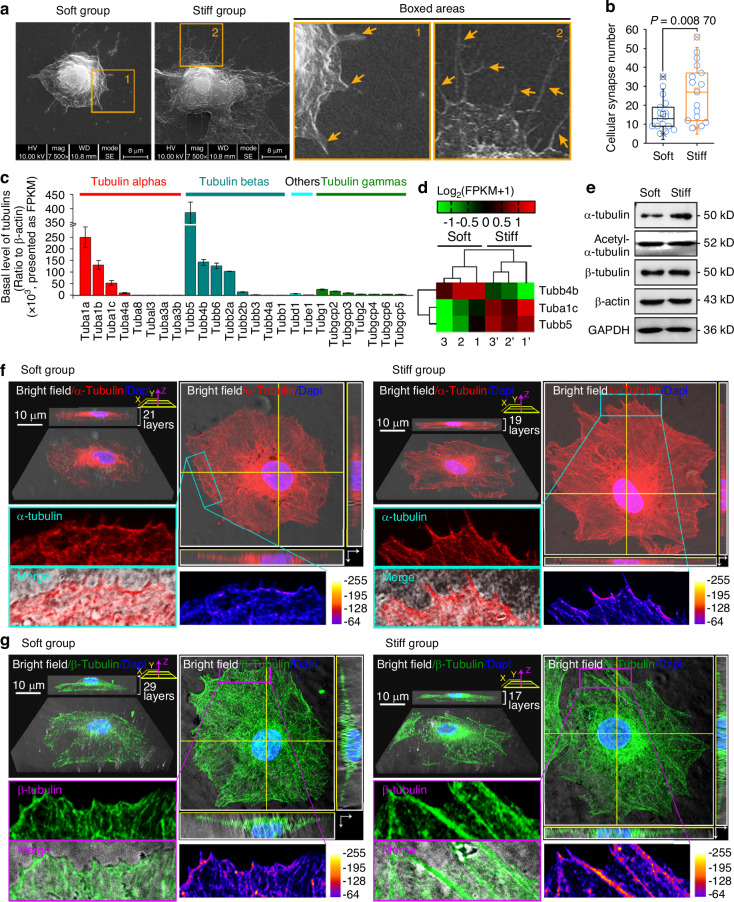
Distribution of tubulin in chondrocytes in response to substrates with different stiffnesses. **a** Representative SEM images showing different cell shapes and cellular synaptic changes in chondrocytes in response to soft and stiff substrates (*n* = 5). Yellow arrows indicate the extension of the cellular synapses. **b** Box and whisker plots indicate cellular synaptic changes in chondrocytes in response to soft/stiff substrates. The results are based on 16 cells from five independent experiments. **c** Histogram based on RNA sequencing showing the basal expression of all tubulin subtypes in chondrocytes. The data are based on FPKM generated by RNA-seq. FPKM, fragments per kilobase of exon model per million mapped fragments. **d** Pheatmap showing the altered subtypes of tubulins in chondrocytes in response to soft/stiff substrates based on RNA-seq. Three pairs of cell samples, i.e., Sample 1 and 1’, Sample 2 and 2’, and Sample 3 and 3’, were obtained from three independent cell isolations (*n* = 3). **e** Western blotting showing the protein-level changes of α-/β-tubulin in chondrocytes in response to soft/stiff substrates. GAPDH and β-actin were used as internal controls (*n* = 3). **f** Representative CLSM images showing the distribution of α-tubulin in chondrocytes in response to soft/stiff substrates. The heatmap (blue-yellow) shows the distribution of α-tubulin in chondrocytes, and gray images show the microtubules at the cell membrane boundary. Cyan arrows indicate microtubule bundles. **g** Representative CLSM images showing the distribution of β-tubulin in chondrocytes in response to soft/stiff substrates. The heatmap (blue-yellow) shows the distribution of β-tubulin in chondrocytes, and gray images show the microtubules at the cell membrane boundary. Purple arrows indicate microtubule bundles

Chondrocytes sense microenvironmental stiffness, spreading over larger surface areas (Fig. 1a and Fig. S1a–c), and produce more cellular synapses on stiff substrates than on soft ones (Fig. 1b), with longer and finer synaptic morphological traits in individual synapses (Fig. S1d). Moreover, chondrocytes seeded onto the stiff substrate exhibited a faster migration rate than those seeded onto the soft substrate (Fig. S2). These microenvironmental stiffness-dependent alterations in cell morphology and motility have been previously attributed to rearrangement of the actin cytoskeleton.^34–36^ To determine whether a similar interdependence exists for microtubules, we first characterized the expression and distribution of proteins in the tubulin family—major components of microtubules. By combining bulk and single-cell RNA sequencing (scRNA-seq) data, we determined the expression profiles of genes in the tubulin family in chondrocytes (Fig. 1c). Among the eight alpha and eight beta tubulin subtypes that are components of microtubules in chondrocytes, seven were highly expressed: Tuba1a, Tuba1b, Tuba1c, Tubb5, Tubb4b, Tubb6, and Tubb2a. Three of these showed significant changes with different substrate stiffnesses (Fig. 1d). Western blotting revealed that the protein expression of α-tubulin was increased in chondrocytes seeded onto the stiff substrate compared to that in chondrocytes seeded onto the soft substrate (Fig. 1e and Fig. S3). To further examine the cytoplasmic distribution of α- and β-tubulins, we performed confocal laser scanning microscopy (CLSM) on individual chondrocytes cultured on soft and stiff substrates. 3D image reconstruction (Fig. 1f, g) revealed that α- and β-tubulins were widely expressed in the cytoplasmic region and distributed along the synaptic regions at the edge of the cell membrane (α-tubulin, boxed cyan areas in Fig. 1f, and β-tubulin, boxed purple areas in Fig. 1g). Heatmaps (flame colors) were used to observe the distribution changes of α-tubulin in Fig. 1f and β-tubulin in Fig. 1g at the cell membrane boundary of chondrocytes. These results indicate a correlation between the distribution of α-/β-tubulins and cell protrusion induced by microenvironmental stiffness.

**Fig. 2:**
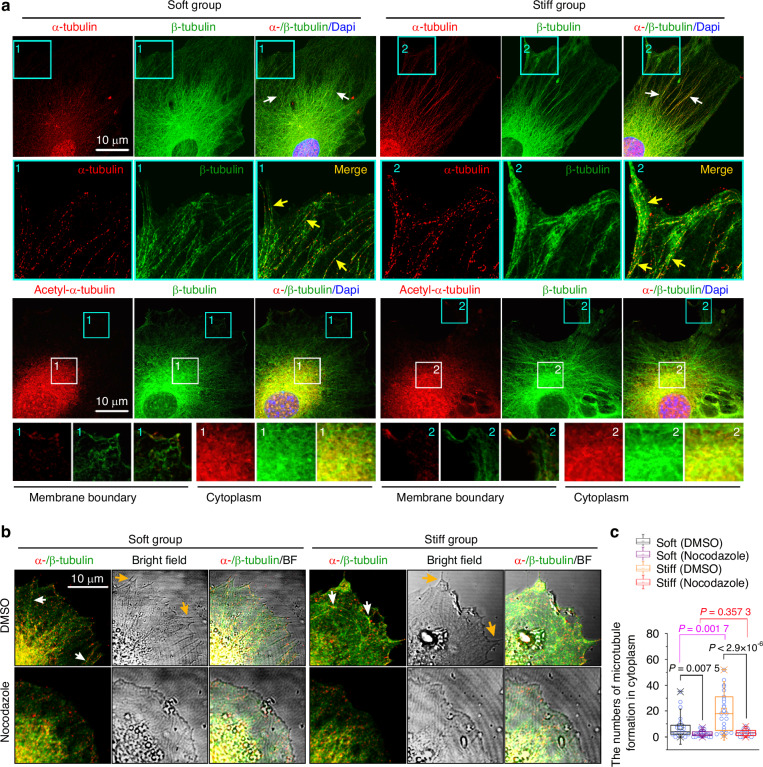
Microtubule distribution is coupled to mechanical stiffness. **a** Representative CLSM images (upper panel) indicating different microtubule morphologies and distributions in chondrocytes in response to soft/stiff substrates (*n* = 8). White arrows indicate different microtubule morphologies in the cytoplasm, and yellow arrows indicate microtubule extension towards the cell membrane boundary. Representative CLSM image (lower) indicating the expression of acetyl-α-tubulin in chondrocytes seeded onto soft/stiff substrates (*n* = 3). The cyan-boxed areas indicate the expression of acetyl-α-tubulin at the boundary of the cell membrane, and white boxed areas indicate the expression of acetyl-α-tubulin in the cytoplasm. **b** Representative CLSM images show that nocodazole disturbs microtubule formation and cellular synaptic extension (*n* = 5). The bright field indicates the cellular synaptic morphology at the cell membrane edge. White arrows indicate microtubule distribution towards the cell membrane boundary and yellow arrows indicate the extension of cellular synapses at the boundary of the cell membrane. **c** Box and whisker plots indicating changes in microtubule number in the cytoplasmic region (**b**). The results are based on 23 cells from five independent experiments

α-tubulin and β-tubulin assemble into linear protofilaments, and 13 such protofilaments combine to form a hollow functional microtubule.^16,37,38^ We next examined microtubule morphological changes in chondrocytes in response to substrate stiffness (Fig. 2a). Microtubules with clearer and more ordered structures were observed on the stiff substrate than on the soft substrate (top two lanes). We also detected the differential distribution of acetylated α-tubulin, a vital regulator in microtubule assembly and function,^39^ in chondrocytes in response to the soft/stiff substrates (lower two lanes). The results showed that at the boundary of the cell membrane, acetylated α-tubulin was expressed following the direction of microtubules, and in the cytoplasmic region near the nucleus, its expression became very high, inferring its potential role in regulating cellular functions such as mechanochemical transduction^40^ and transcriptional activity.^41^ To further investigate the interdependence between microtubules and microenvironmental stiffness, we first used nocodazole, a chemical inhibitor that binds to β-tubulin and disrupts microtubule assembly and disassembly (Fig. 2b). Treatment with 200 nmol/L nocodazole reduced the assembly of microtubules in both soft and stiff groups (Fig. 2b, lower panel fluorescence); quantification of microtubule numbers confirmed this change (Fig. 2c). Importantly, nocodazole treatment also induced the reduction in cell protrusions and synapses (yellow arrows indicated in the bright fields), which were distinct from those in the DMSO controls. At protein levels, we found that nocodazole did not alter the total protein levels of α- or β-tubulin in chondrocytes but partially reduced the expression level of acetylated α-tubulin (Fig. S4). This inhibitor experiment indicates that microtubule assembly (not the expression levels of α- or β-tubulin) is necessary for the protrusion or synapse spreading of chondrocytes. Moreover, we used blebbistatin, an inhibitor of myosin II, to block mechanosensation and found that chondrocytes showed no cellular protrusions or synapses after 25 μmol/L blebbistatin treatment (Fig. S5a, bright fields in both α-tubulin, upper, and β-tubulin, lower) and that the expression of bundle-like α-tubulins (upper panel, red) and β-tubulins (lower panel, green) at the edge of the cell membrane was largely reduced, suggesting decreased microtubule formation or polymerization-depolymerization kinetics. However, total expression of α- or β-tubulin at gene and protein levels were shown to be unchanged in chondrocytes on the soft/stiff substrates treated with/without blebbistatin (Fig. S6). Quantification of the bundle-like number in α-tubulins and β-tubulins further confirmed these results (Fig. S5b).

Taken together, these results suggest that microenvironmental stiffness affects the tubulin cytoskeleton, which in turn partially determines the cell morphology and microstructure.

### Mechanical stiffness mediates microtubule distribution in mitotic chondrocytes

**Fig. 3:**
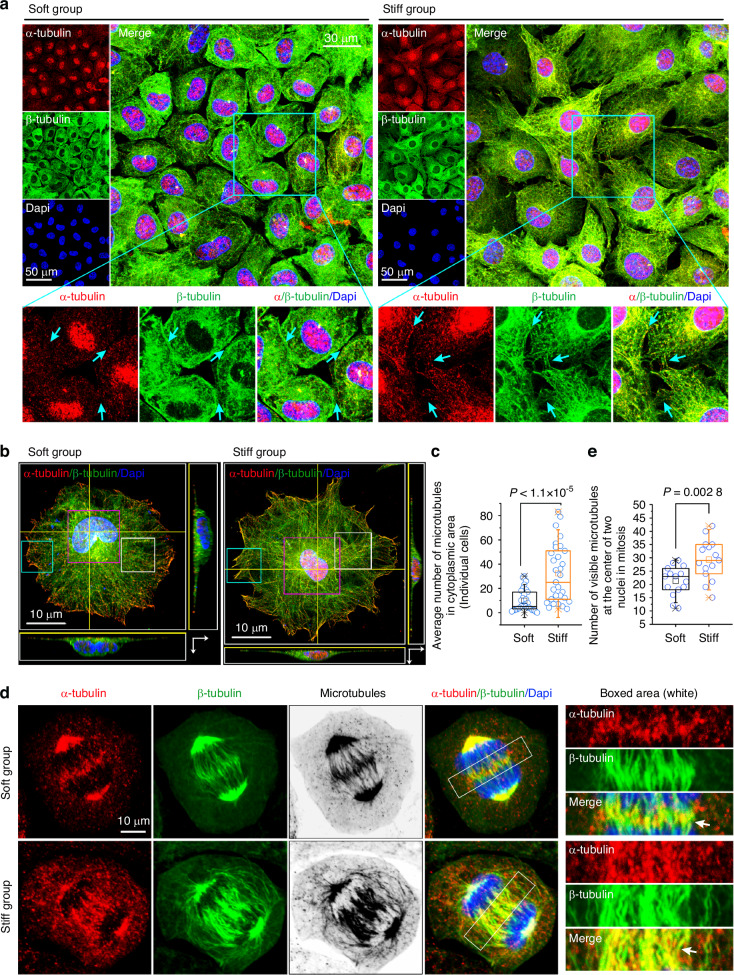
Changes in microtubule bundles in mitotic chondrocytes in response to substrates with different stiffnesses. **a** Representative CLSM images showing changes in microtubule bundles in chondrocytes in response to soft/stiff substrates. The cyan arrows indicate microtubule connections between the two cells. **b** Top-view CLSM images showing morphological changes in the microtubules of individual chondrocytes in response to soft/stiff substrates. **c** Quantitative analysis of the total number of microtubules in the cytoplasmic region showed that more microtubule bundles were formed on the stiff substrate than on the soft substrate. The results are based on 30 cells from five independent experiments. **d** Representative CLSM images showing the distribution of microtubules in mitotic chondrocytes in response to soft or stiff substrates (*n* = 5). Boxed areas (right) indicate changes in microtubule bundles at the center of the two nuclei. White arrows indicate different morphologies and distributions of microtubule bundles in the center of the two immature nuclei at anaphase. **e** Quantitative analysis of the number of microtubule bundles at the center of the two nuclei in mitotic chondrocytes. The results are based on 15 cells from five independent experiments

Next, we explored microtubule changes in chondrocytes at the quiescent state (G0) stage (the stage at which most chondrocytes exist) induced by soft/stiff substrates. Chondrocyte microtubules formed intracellular junctions when cells were seeded at a relatively high density. Microtubule staining of cells that reached ~90% confluence revealed different distribution patterns of α- and β-tubulin in chondrocytes (Fig. 3a). α-Tubulin showed high accumulation in the nuclear region, while β-tubulin was mostly distributed in the cytoplasm in both soft and stiff groups. However, when more α-tubulin was distributed in the cytoplasm of cells on the stiff substrate, chondrocytes formed more and clearer microtubule bundles than those on the soft substrate. The connection of microtubules formed between two adjacent chondrocytes (cyan arrows) indicated that microtubules may play various roles in cell-to-cell junctions and communication in response to different stiffnesses.^42^ To further observe the changes in the microtubules of individual chondrocytes cultured on soft/stiff substrates, we used CLSM layer scanning and 3D image reconstruction (Fig. 3b and Fig. S7a). Microtubules (merged images of α-tubulin and β-tubulin, yellow) in the soft group were observed to be shorter, presenting discontinuous tubule-like structures relative to those in the stiff group. Quantitative analysis revealed a change in the number of microtubules in chondrocytes cultured on soft/stiff substrates (Fig. 3c). Detailed analyses of microtubule changes (Fig. S7b–g) showed that the cell height of the seeded chondrocytes in the soft group was higher than that in the stiff group after 12 h (Fig. S7b). We characterized the morphological changes in the microtubules in the nuclear, cytoplasmic, and plasma membrane regions (Fig. S7c–g). A considerable number of microtubules were observed in the nuclear region, spanning the entire nucleus on the stiff substrate, with stronger fluorescence intensities for both α-tubulin (red) and β-tubulin (green) in the same regions (Fig. S7c). Linear quantification of α-tubulin confirmed the accumulation of microtubules in the nuclear region (Fig. S7d), and the total fluorescence intensity of the nuclear regions (per cell) in the stiff group was significantly stronger than those in the soft group (Fig. S7e). Similar findings were observed in the cytoplasmic (Fig. S7f) and membrane regions (Fig. S7g), with more and larger microtubules on the stiff substrate. Notably, not all chondrocyte nuclei exhibited the aggregation of α-tubulin in a quiescent state (G0) (Fig. S8a); however, the proportion of chondrocytes with nuclear aggregation of α-tubulin was significantly higher for the stiff substrate than for the soft substrate (Fig. S8b). The result that a higher expression of α-tubulin aggregated in the nuclei of chondrocytes on the stiff substrate might explain the increase in α-tubulin in chondrocytes on the stiff substrates upon western blotting (Fig. 1e).

We extended our study to include cells undergoing mitosis. Western blotting revealed that the expression of cyclin B1, cyclin B2, and CDK1 increased in chondrocytes in response to stiff substrates relative to those in response soft substrates (Fig. S9), suggesting that chondrocytes may accelerate their cell cycle when induced by a stiffer substrate. During prometaphase, spindle-shaped microtubules began to form and were more strongly expressed in chondrocytes on the stiff substrate (Fig. S10a). At anaphase—when independent offspring chromatin aggregated but did not form a complete circular nucleus, we found that the distribution of microtubules showed the most significant differences in chondrocytes in response to soft/stiff substrates (Fig. 3d). The number, length, and size of microtubules between the two immature nuclei were greater in chondrocytes on the stiff substrate than in those on the soft substrate (Fig. 3d, white arrows). Quantitative analysis of the number of visible microtubules between two immature nuclei confirmed this result (Fig. 3e).

Between anaphase and telophase, when round, mature offspring nuclei formed but cytoplasmic separation had not yet occurred (Fig. S10b), microtubules were distributed throughout the cytoplasm in both the soft and stiff groups; however, in the stiff substrate, the arrangement of microtubules became increasingly ordered, extending around the nucleus. At the middle point between the two nuclei, the concentration of microtubules was the highest, and there were flake-like highlights of microtubules in chondrocytes on the stiff substrate but dot-like highlights in those on the soft substrate. The highlighted area on the stiff substrate was much larger than that on the soft substrate (indicated by the white arrows). At the final stage of cell division, as microtubules connecting the two progeny cells were segmented and disrupted, we observed morphological changes in the microtubule bundles of the chondrocytes in response to the substrate stiffness (Fig. S10c, d). When the microtubule bundles between the two progeny cells were disconnected, the β-tubulin component disappeared first (Fig. S10c, white arrows), which caused the microtubule bundle to resemble a node that was cut off at this site. In addition, the microtubule bundles in chondrocytes seeded on the soft substrate were approximately twice as large as those in chondrocytes seeded on the stiff substrate (Fig. S10d, both fluorescent bundles, left; size quantification, right).

Collectively, these results indicated that chondrocytes can form more and longer microtubules to participate in cell division.

### Refilin B is a vital mediator of microtubule formation in chondrocytes in response to mechanical stiffness

**Fig. 4:**
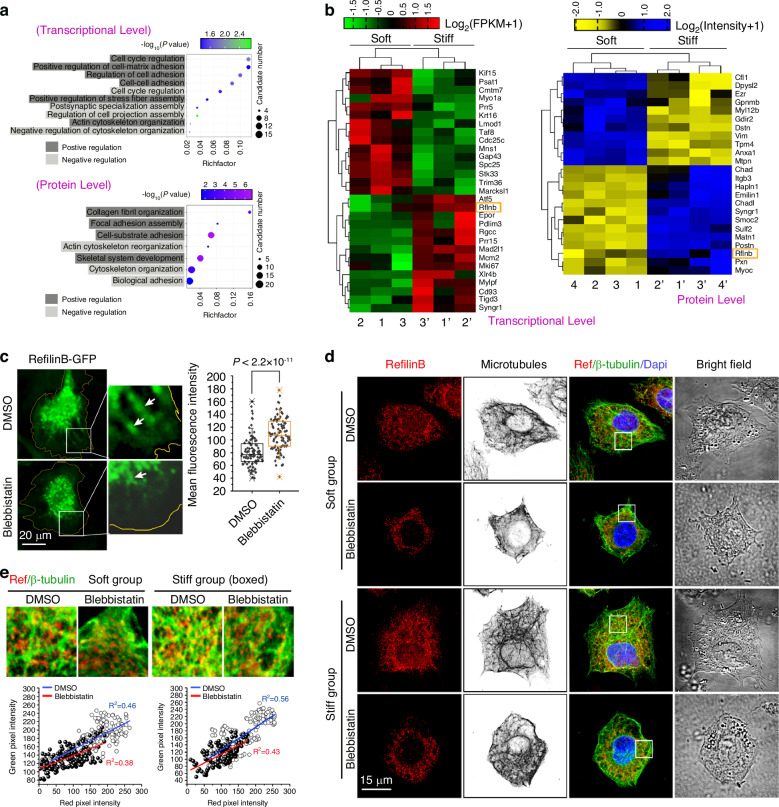
Refilin B correlates with the microtubule changes in chondrocytes in response to substrates with different stiffnesses. **a** GO analysis showing biological process (BP) changes related to chondrocyte microtubules in response to soft/stiff substrates by bulk RNA-seq (transcriptional level, upper) and protein mass spectrometry (protein level, lower). **b** Pheatmap showing changes in gene and protein candidates related to microtubules in response to soft/stiff substrates. The left panel shows gene candidates based on bulk RNA-seq, and the right panel shows protein candidates based on MS. **c** Overexpression of refilin B-GFP in chondrocytes cultured on Petri dishes treated with blebbistatin. Left, representative images of chondrocytes overexpressing refilin B-GFP Right, box, and whisker plots showing the mean fluorescence intensity of refilin B in chondrocytes with or without blebbistatin treatment. The data were calculated using 90 cells (per group) from five independent experiments. White arrows indicate the different distributions of refilin B with or without blebbistatin at the boundary of the cell membrane. **d** Representative CLSM images showing the cytoplasmic distribution of refilin B and microtubules in chondrocytes cultured on soft/stiff substrates with or without blebbistatin treatment. **e** Correlation analysis of the distribution of refilin B and microtubules in the peripheral cytoplasm of chondrocytes cultured on soft/stiff substrates with or without blebbistatin treatment. Blue linear fitting demonstrates the correlation between refilin B (red pixel intensity) and β-tubulin (green pixel intensity) at the boundary of the cell membrane on the soft (left) and stiff (right) substrates, and red linear fitting demonstrates the correlation between refilin B (red pixel intensity) and β-tubulin (green pixel intensity) at the boundary of the cell membrane on the soft (left) and stiff (right) substrates after blebbistatin treatment

To identify potential mediators of microtubule formation induced by mechanical stiffness, we performed bulk RNA sequencing and mass spectrometry on chondrocytes cultured on both soft and stiff substrates. Gene Ontology (GO) analysis of the differentially expressed genes and proteins revealed that, at the transcriptional and proteomic levels, all the altered mediators involved in microtubule regulation were involved in biological processes such as cell adhesion, cell-matrix interactions, cytoskeleton rearrangement, cell synaptic orientation, and the cell cycle (Fig. 4a). Comparison of the mediators at the mRNA and protein levels revealed the following common mediators (Fig. 4b): synaptogyrin-1 (Syngr1), a cellular non-neuronal paralog of the synaptic vesicle protein,^43^ and refilin B (Rflnb), an indispensable actin—and tubulin—cytoskeleton mediator,^44,45^ suggesting that these two proteins may mediate microtubule organization induced by mechanical stiffness. To further confirm these findings at the single-cell level, we performed scRNA-seq based on 18 000 cells per group and identified the mediator, Rflnb. Uniform manifold approximation and projection (UMAP) showed the levels of Rflnb in chondrocyte subsets in response to soft/stiff substrates (Figure S11a), and we verified its total gene expression in all chondrocyte subsets (Figure S11b). A heatmap was generated to show the average gene expression of Rflnb in chondrocytes in response to substrate stiffness (Fig. S11c). At the protein level, differences in the expression of Rflnb were confirmed by western blotting (Fig. S11d).

To determine the mechanical response characteristics of Rflnb, we first overexpressed it in chondrocytes using a GFP-Rflnb lentiviral plasmid. After pretreatment with blebbistatin, the GFP fluorescence intensity in the chondrocytes decreased, and the GFP fluorescence that originally extended toward the cell membrane boundary disappeared after treatment with blebbistatin (Fig. 4c, white-boxed areas). Quantitative analysis confirmed a reduction in GFP fluorescence in individual chondrocytes in the blebbistatin-treated group relative to those in the normal group (Fig. 4c, right). To confirm the mechanical response characteristics of endogenous Rflnb, we detected its expression in chondrocytes cultured on soft/stiff substrates treated with blebbistatin using immunofluorescence (Fig. 4d, e). We found that the expression of Rflnb decreased, and its distribution in the cellular membrane extension area decreased in the blebbistatin-treated groups relative to the non-treated soft/stiff groups. Simultaneously, the number of intracellular microtubules and the area of chondrocyte cell spreading decreased (Fig. 4d). We correlated the expression of Rflnb and bundle-like β-tubulin in chondrocytes cultured on soft/stiff substrates treated with blebbistatin (Fig. 4e) and found that blebbistatin reduced the expression of both Rflnb and bundle-like β-tubulin at the microtubule formation region (linear correlations were reduced in chondrocytes on the soft/stiff substrate upon blebbistatin treatment).

**Fig. 5:**
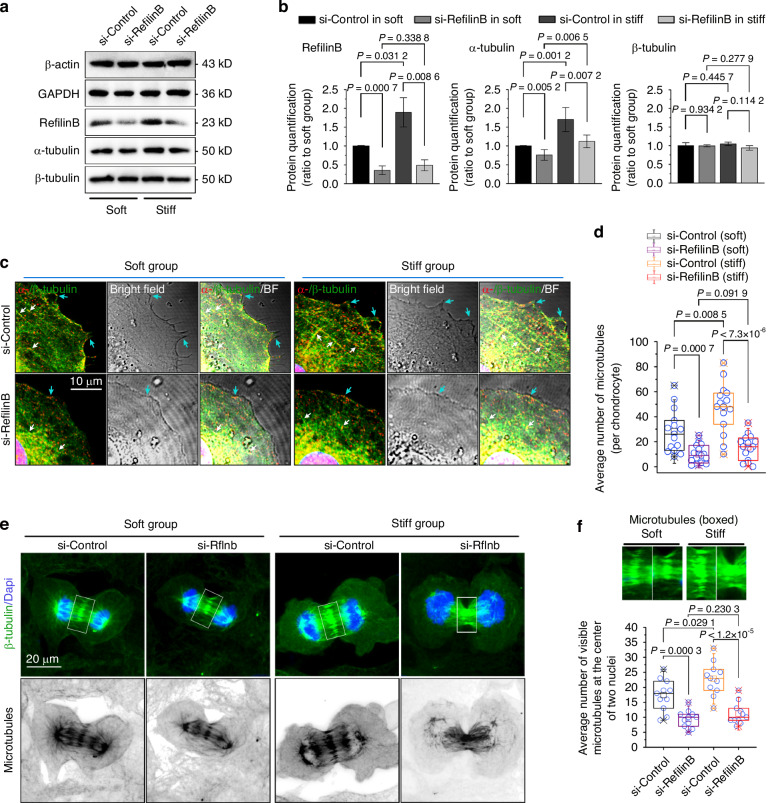
Refilin B knockdown impairs microtubule formation in chondrocytes in response to substrates with different stiffnesses. **a** Western blot showing the protein changes of tubulins in chondrocytes by si-refilin B in soft/stiff substrates. **b** Quantitative analysis of refilin B and tubulin (**a**). Target proteins were quantified as a ratio to β-actin. Data are obtained from three independent experiments (*n* = 3). **c** Representative CLSM images showing the correlation between microtubule formation and cell spreading of individual chondrocytes on soft/stiff substrates by si-refilin B (*n* = 4). White arrows indicate microtubule morphology in the cytoplasm and cyan arrows indicate microtubule extension along the cell membrane boundary. **d** Quantitative analysis of microtubule number in the cytoplasm of individual chondrocytes. Results are based on 15 cells from four independent experiments. **e** Representative CLSM images showing microtubule changes in mitotic chondrocytes on soft/stiff substrates after si-refilin B treatment (*n* = 4). **f** Quantitative analysis of microtubule numbers at the center of the two nuclei in mitotic chondrocytes (boxed areas in (**f**)). The results were obtained from 11 cells in four independent experiments

To further demonstrate the influence of Rflnb on microtubule formation in mitotic chondrocytes, we used siRNA interference to knockdown Rflnb. After determining the knockdown efficiency of Rflnb (Fig. S12), we first determined that Rflnb knockdown had an impact on chondrocyte migration on both soft and stiff substrates (Fig. S13). We then focused on microtubule changes in chondrocytes cultured on soft/stiff substrates. By western blotting, we found that Rflnb knockdown reduced the protein expression of α-tubulin but not β-tubulin in chondrocytes cultured on the soft/stiff substrates (Fig. 5a), and quantitative analysis confirmed the protein-level changes in Rflnb and α-tubulin (Fig. 5b). Moreover, Rflnb knockdown decreased the gene expression of α-tubulin in chondrocytes cultured on the soft/stiff substrates (Fig. S14). Using immunofluorescence, we showed that Rflnb knockdown reduced the formation of microtubules in chondrocytes cultured on both soft and stiff substrates (Fig. S15). To further investigate the changes in microtubules and cell protrusions/synapses induced by si-Rflnb, we performed CLSM layer scanning (Fig. 5c). The results showed that si-refilin B treatment significantly decreased the number of cytoplasmic microtubules in chondrocytes and the number of cell protrusions/synapses. Quantitative analysis confirmed the reduction in microtubule formation in individual chondrocytes cultured on soft/stiff substrates (Fig. 5d). We further detected the influence of Rflnb on microtubule formation in mitotic chondrocytes. Using immunofluorescence, we found that the most significant effect of Rflnb knockdown was a decrease in the number of microtubules that appeared at the center of the two nuclei at anaphase (Fig. 5e). Quantitative analysis confirmed this observation (Fig. 5f).

Taken together, these results indicated that Rflnb acts as a vital mediator of microtubule formation, targeting α-tubulin in chondrocytes in response to mechanical stiffness.

### Rflnb-mediated microtubule formation requires the participation of p-Smad3 signaling

**Fig. 6:**
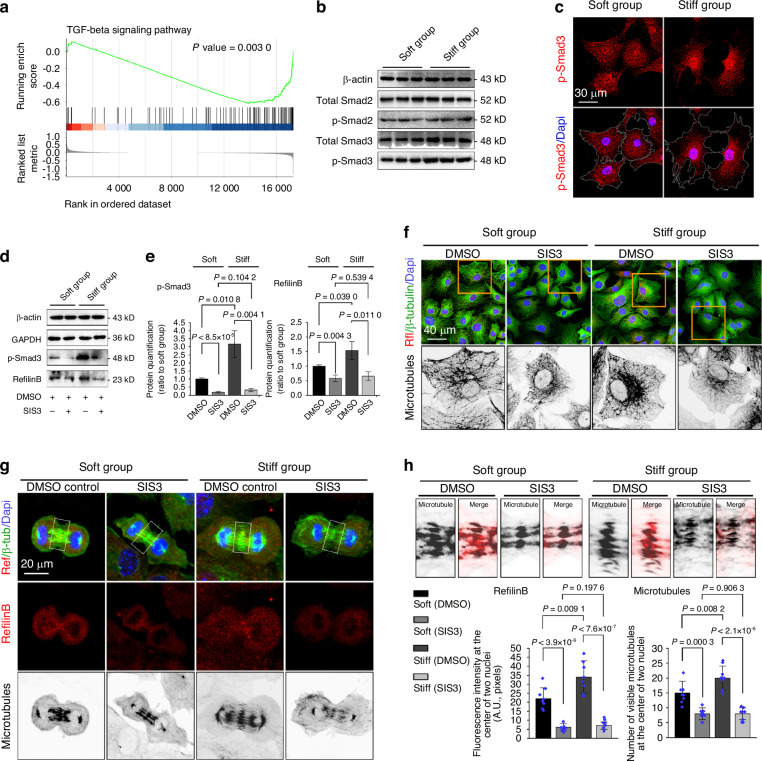
Refilin B mediates microtubules through p-Smad3 signaling in chondrocytes in response to substrates with different stiffnesses. **a** Enrichment plots from gene set enrichment analysis (GSEA) showing TGF-beta signaling is involved in matrix stiffness-mediated chondrocytes (single-cell RNA-seq). **b** Western blot showing protein changes in total and phosphorylated samd3 in chondrocytes on soft/stiff substrates. **c** Representative CLSM images showing the nuclear accumulation of p-Smad3 in chondrocytes on soft/stiff substrates. **d** Western blotting showing protein changes of p-Smad3 and refilin B in chondrocytes on soft/stiff substrates after treatment with the inhibitor SIS3. **e** Quantitative analysis of p-Smad3 and refilin B in chondrocytes in response to soft/stiff substrates following SIS3 treatment (**d**). Target proteins were quantified as a ratio to β-actin. Data are based on three independent experiments (*n* = 3). **f** Representative CLSM images showing changes in microtubules in a collection of chondrocytes on soft/stiff substrates following SIS3 treatment. The boxed areas (yellow) indicate changes in chondrocyte microtubules (*n* = 4). **g** Representative CLSM images showing changes in refilin B and microtubules in mitotic chondrocytes on soft/stiff substrates after SIS3 treatment (*n* = 3). **h** Quantitative analysis of refilin B and microtubule numbers at the center of the two nuclei in mitotic chondrocytes (boxed areas in (**g**)). The results are based on seven cells from three independent experiments

Rflnb is reported to be a downstream effector of TGF-β signaling.^46,47^ It can compete with Smad2 and Smad3, promoting their release into the cytoplasm.^44,48^ It can also bind to Smad3 and enhance the accumulation of p-Smad3 in the nucleus, thereby promoting its interaction with transcription factors to activate or repress gene transcription.^49^ We examined endogenous TGF-β signaling in chondrocytes in response to mechanical stiffness and found significant changes in this signaling pathway by GSEA (Fig. 6a, *P* = 0.003). Based on the changes in Smad genes in this gene set, we identified other relevant signaling pathways, including the Hippo, Wnt, and signaling pathways, that regulate the pluripotency of chondrocyte precursors, in which Smads were also enriched (Fig. S16). Among these gene sets, we detected changes in the expression of Smad3, but not Smad2 (Fig. S17). At the protein level, we investigated the changes in Smad2 and Smad3 by western blotting and found that the total Smad3 and p-Smad3 levels were higher in the stiff group than in the soft group (Fig. 6b). Moreover, the change in phosphorylation in chondrocytes cultured on soft/stiff substrates was much greater than the change in total Smad3 levels (Fig. S18). Immunofluorescence analysis revealed that the nuclear accumulation of p-Smad3 in the stiff group was greater than that in the soft group (Fig. 6c and Fig. S19).

To determine the role of p-Smad3 in Rflnb-mediated microtubules, we applied SIS3, a specific Smad3 inhibitor that selectively inhibits Smad3 signaling.^50^ Western blotting revealed that SIS3 effectively inhibited the expression of p-Smad3, and that the expression of Rflnb correspondingly decreased in chondrocytes cultured on soft/stiff substrates (Fig. 6d). Quantitative analysis of p-Smad3 and Rflnb confirmed these changes (Fig. 6e). Western blotting also showed that SIS3 decreased the expression of α-tubulin but not β-tubulin in chondrocytes seeded on the soft/stiff substrates (Fig. S20). Using immunofluorescence, we detected changes in the number of microtubules in a cohort of chondrocytes in response to mechanical stiffness (Fig. 6f). The results showed that endogenous inhibition of p-Smad3 signaling decreased the expression of Rflnb and largely reduced the number of microtubules in chondrocytes cultured on both soft and stiff substrates. To further investigate the changes in microtubules and cell protrusions/synapses, we applied CLSM layer scanning to individual chondrocytes (Fig. S21a). The results indicated that as the amount of Rflnb decreased in the cytoplasm, the number of microtubules (bundle-like β-tubulin) greatly decreased, and the number of cell protrusions/synapses was decreased in chondrocytes cultured on the soft and stiff substrates after SIS3 treatment. Quantitative analysis confirmed the decrease in Rflnb levels and the reduction in microtubule (Fig. S21b) and cellular synapse numbers (Fig. S21c) in individual chondrocytes cultured on soft/stiff substrates treated with SIS3. Next, we detected changes in Rflnb and microtubules in chondrocytes during anaphase. Immunofluorescence revealed a significant decrease in the number of microtubules at the center of the two nuclei during anaphase (Fig. 6g, white boxes). A decrease in the number of microtubules was accompanied by a decrease in Rflnb expression in the same region. Detailed analysis of the correlation between Rflnb expression (red) and microtubule formation (black) at the center of the two nuclei confirmed this change during anaphase (Fig. 6h, upper). Quantitative analysis confirmed a decrease in Rflnb expression and a decrease in the number of microtubules at the center of the two nuclei (Fig. 6h, lower). In addition, to detect the interdependence between Rflnb and p-Smad3, we performed immunofluorescence analysis to investigate the expression of p-Smad3 in chondrocytes after Rflnb silencing (Fig. S22). The results revealed that silencing of Rflnb significantly enhanced the nuclear translocation of p-Smad3.

Collectively, these data indicated that p-Smad3 signaling is important for Rflnb-mediated microtubule formation.

### Integrin-linked kinase (ILK) is required for Rflnb-mediated microtubule formation in chondrocytes in response to mechanical stiffness

**Fig. 7:**
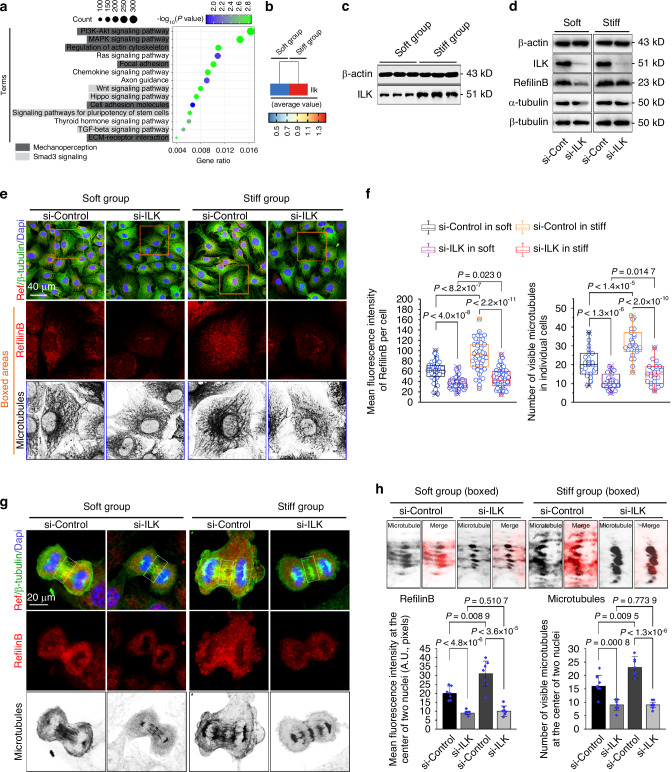
ILK is required for refilin B-mediated microtubule formation in chondrocytes in response to substrates with different stiffnesses. **a** KEGG analysis showing the signaling pathways related to mechanoperception in chondrocytes in response to substrates with different stiffnesses. **b** Pheatmap showing the average gene expression of ILK by single-cell RNA-seq in chondrocytes in response to soft/stiff substrates. **c** Western blot showing protein changes in ILK in chondrocytes in response to soft/stiff substrates. Images were obtained from three independent experiments (*n* = 3). **d** Western blot showing changes in refilin B and tubulin in chondrocytes on soft/stiff substrates after si-ILK treatment. **e** Representative CLSM images showing changes in refilin B and microtubules in chondrocytes grown on soft/stiff substrates with si-ILK. The boxed areas (yellow) indicate changes in refilin B and microtubules in the chondrocytes (*n* = 5). **f** Quantitative analysis of refilin B (left) and microtubules (right) in chondrocytes grown on soft/stiff substrates using si-ILK (**g**). The mean fluorescence intensity of refilin B in chondrocytes was based on 41 cells from 5 independent experiments, and the visible microtubule number was based on 23 cells from 5 independent experiments. **g** Representative CLSM images showing changes in refilin B and microtubules in mitotic chondrocytes on soft/stiff substrates induced by si-ILK (*n* = 5). **h** Quantitative analysis of refilin B and microtubule numbers at the center of two nuclei in mitotic chondrocytes (boxed areas in **g**). The results are based on seven cells from five independent experiments

To determine the relationship between Rflnb and mechanoperception/mechanotransduction in chondrocytes, we analyzed the signaling pathways activated in response to mechanical stiffness. We clustered the signaling pathways most relevant to mechanoperception/mechanotransduction by conducting a KEGG analysis of the scRNA-seq data (Fig. 7a) and identified the top six signaling pathways related to mechanoperception: ECM-receptor interaction, focal adhesion, cell adhesion molecules, cytoskeleton regulation, PI3K-Akt, and MAPK signaling (Fig. 7a and Fig. S23), in addition to Smad3-related signaling. Importantly, we investigated the genes enriched in these pathways and found that the integrin protein family—a typical signal receptor for mechanoperception/mechanotransduction^51^—was widely involved in the above signaling pathways. The members include Itgal, Itga4, Itga5, Itga6, Itgav, Itgam, Itgb2, and Itgb5 (Fig. S24a). Given that ILK has been shown to play an important role as an adapter in the activation of downstream integrin signaling, including focal adhesion formation and mechanical-chemical transduction,^52,53^ we examined its expression and found that ILK was significantly upregulated in chondrocytes cultured on stiff substrates compared to those cultured on soft substrates (Fig. 7b). At the protein level, we detected its differential expression in chondrocytes cultured on soft and stiff substrates by western blotting (Fig. 7c and Fig. S25a). Moreover, we used the online STRING database to establish the correlation between ILK and mechanosensitive integrins in chondrocytes in response to substrate stiffness (Fig. S24b), which provides evidence similar to previous reports revealing their relationship^54,55^

To test whether ILK could regulate Rflnb, we applied siRNA interference to knockdown ILK. By western blotting, we found that ILK knockdown significantly reduced the expression of Rflnb in chondrocytes cultured on the soft/stiff substrates. Moreover, ILK knockdown decreased the expression of α-tubulin but not β-tubulin (Fig. 7d and Fig. S25b). Additionally, ILK knockdown partially reduced the expression of phosphorylated Smad3 (Fig. S26), inferring there might be mutual regulatory mechanisms between them. To determine the effect of ILK knockdown on microtubule formation, we used immunofluorescence to detect changes in mitotic chondrocyte microtubules (Fig. 7e). CLSM revealed that ILK knockdown not only reduced the expression of Rflnb (red) but also impaired microtubule formation in chondrocytes cultured on both soft and stiff substrates. These results were further confirmed by quantitative analyses of the average fluorescence intensity of Rflnb and the number of visible microtubules in individual chondrocytes (Fig. 7f). In mitotic chondrocytes, we observed substantial changes in the number of microtubules at the center of the two nuclei at anaphase on both soft and stiff substrates (Fig. 7g). At the same time, the expression of Rflnb in this region was significantly reduced (red). To further show the correlation between changes in the expression of Rflnb and changes in the number of microtubules at the centers of two nuclei, we boxed and enlarged these areas in Fig. 7h. The results showed that ILK knockdown caused a decrease in the expression of Rflnb and a decrease in the number of microtubules in these regions.

Overall, we established a correlation between Rflnb-mediated microtubule formation and mechanical stiffness.

## Discussion

Cartilage is a connective tissue without blood vessels, lymphatic vessels, and nerves, and is composed solely of resident chondrocytes and ECM, mainly composed of type II collagen and proteoglycans, which work to resist the mechanical load received by cartilage.^56,57^ Chondrocytes within the lacunae maintain their capacity for ECM synthesis and turnover, with low mitotic activity.^58^ However, in joint diseases such as OA, the stiffness of the cartilage ECM microenvironment undergoes significant alterations, particularly becoming much stiffer in late-stage OA.^59^ Intriguingly, studies have suggested that chondrocytes exhibit increased metabolic and synthetic activities during early OA to counteract damage.^60,61^ Exploring the response of chondrocytes to mechanical stimulation is crucial, not only because chondrocytes are typical mechanosensing cells, but also because of the great challenge of cartilage repair in diseases such as trauma and OA.^62–64^ Articular cartilage is subjected to constant mechanical stresses of different intensities. While moderate mechanical loading produced by normal joint movement is beneficial to the health of articular cartilage, local mechanical overload leads to excessive mechanical compression of chondrocytes, weakened homeostasis of the cartilage microenvironment, and reduced cartilage thickness, which are detrimental to articular cartilage and may ultimately contribute to its pathogenesis.^65,66^ Chondrocytes, as mechanically sensitive cells, can respond to mechanical stress and synthesize extracellular components, including type II collagen, aggrecan, hyaluronic acid, and chondroitin sulfate, thus facilitating homeostasis of the ECM network.^67–69^ Previous studies have elucidated the regulation of microenvironmental mechanics on the actin cytoskeleton (actin microfilament) and its related mechanically sensitive protein clusters, such as the integrin family, focal adhesion plaque, and RhoA/ROCK signaling,^36,70–72^ but much less is known about the tubulin cytoskeleton (tubulin microtubule). In the current study, we characterized the changes in microtubules and the corresponding regulatory mechanisms in chondrocytes induced by two distinct types of microenvironment stiffness: a soft substrate with a Young’s modulus of ~46 kPa, which approximates the stiffness of the normal ECM of chondrocytes, and a stiff substrate with a Young’s modulus of ~450 kPa, which is equivalent to the stiffness in the local chondral sclerotic zone during severe OA.^33,73^ These results help us understand the mechanical regulation of microtubules and their role in chondral pathogenesis, and provide an in-depth understanding of biophysical–biochemical changes, revealing a potent cue for cartilage remodeling and OA therapy.

Several studies have shown that changes in the components, structure, and morphology of the cytoskeleton are involved in various kinds of mechanotransduction.^74,75^ The chondrocyte cytoskeleton consists of actin fibers (actin filaments), microtubules, and intermediate vimentin filaments. Among them, actin fibers are well-recognized to change their structure and morphology under mechanical stimulation. Campbell et al. indicated that compressive strain can promote cortical actin depolymerization in chondrocytes.^76^ Xu et al. showed that cyclic tensile strain forces chondrocytes to reorganize actin stress fibers.^77^ Knight et al. found that hydrostatic pressure can also reorganize actin stress fibers in chondrocytes.^78^ Our group previously revealed that static mechanical perception induces actin filament reorganization in chondrocytes and alters their phenotype^70,72^ and cross-talk.^79^ Moreover, Sarasa-renedo et al. reported that the formation of actin stress fibers required the activation of the Rho GTPase family member Rho A.^80^ For microtubule cytoskeleton, some reports have shown the influence of mechanical loading on the expression of the subunit β-tubulin in chondrocytes.^81,82^ However, there have been no characterizations of microtubules in response to stiffness. Here, we first indicated that microtubules are mechanosensitive in chondrocytes, characterized the different structures and morphologies of microtubules in chondrocytes in response to substrate stiffness, and identified all the gene candidates that may be involved in this response.

We identified Rflnb as a vital mediator responsible for chondrocyte microtubule assembly in response to substrate stiffness. Refilins, including refilinA and Rflnb, are known filamin-binding proteins that interact closely with various actin proteins and affect the final formation of actin bundles.^44,47^ Mice with knockout of a single refilin do not show any phenotypic changes, but those with double refilin knockout show a skeletal malformation that is similar to that observed in filamin-deficient mice.^44,46,83^ However, refilin A and Rflnb are considered to have clear structural and functional differences. For instance, refilinA and Rflnb show approximately 40% identity and 48% similarity in *Rattus norvegicus*^84^; due to their instability, the mechanisms that regulate their expression levels are also different.^83^ Nishiyama et al. reported that the regulation of refilin A was dependent on the transcriptional level, while that of Rflnb was due to enhanced protein stability and accumulation.^85^ Refilins are downstream TGF-β effectors and participate in the formation of flnb-Smad3 complex; Rflnb shows a strong interaction with Smad3 signaling and can relieve chondrocyte hypertrophy and apoptosis.^49^ In the current study, we showed that the interaction between Rflnb and Smad3 signaling was also mediated by mechanical stiffness. The basal expression levels of Rflnb and Smad3 in chondrocytes in response to the soft and stiff substrates were significantly different (Figs. 4a, b and 6b, c), and the inhibition of endogenous Smad3 signaling eliminated the differential expressions of Rflnb on soft and stiff substrates (Figs. 6d–f), indicating the regulatory role of Smad3 signaling in Rflnb action and thus in microtubule dynamics in the mitotic cell cycle of chondrocytes. Although Smad2/3 often functions as a complex that translocates into the nucleus to regulate target genes, there are significant functional differences between Smad2 and Smad3. For example, Smad3 can directly recognize specific DNA sequences,^86^ whereas Smad2 requires auxiliary factors, such as a mixed-like endodermal regulator (mixer), to indirectly regulate gene expression.^87^ Combined with the results in Fig. 6b, Figs. S18 and S19, we inferred that Smad2 plays a certain role in the microtubules regulated by Rflnb, but it is not as dominant as Smad3. Moreover, on a stiff substrate, other Smad genes, including Smad4/5/6/7, were upregulated in chondrocytes (Fig. S17a). These members may also be involved in regulating microtubules triggered by Rflnb, even though these regulatory pathways may be indirect or auxiliary owing to their relatively low expression levels (Fig. S17b) and special functions in chondrocytes. For instance, studies have revealed that Smad4 functions as a complex with Smad3 and helps activate Smad3 signaling to regulate downstream gene transcription, rather than independently exerting regulatory functions on chondrocytes.^87^ Smad5/6/7 is primarily involved in antagonizing BMP signaling and mediating negative feedback regulation.^88,89^ Their enhanced expression on stiff substrates likely reflects compensatory paracrine signaling triggered by mechanical cues rather than directly driving chondrocyte mitosis.

To further validate the importance of Smad3 signaling in regulating polymerization–depolymerization kinetics of microtubules in chondrocytes, we found that the expression of α-tubulin protein was reduced in chondrocytes after being treated with the inhibitor SIS3 (Figure S20a). Rflnb knockdown diminished both α-tubulin expression and microtubule formation, as shown in Fig. 5. Taken together, these findings demonstrate that both Smad3 signaling and Rflnb have the same effect on the expression of α-tubulin in chondrocytes, indicating the importance of the Rflnb–Smad3 complex in microtubule kinetics. The expression profiles of Rflnb (http://biogps.org) revealed that it is highly expressed in bone-related cells (e.g., chondrocytes and osteoblasts), besides the fact that Rflnb is critically involved in skeletal mechanosensing^90^ and acts as a downstream effector of TGF-β signaling.^46,47^ We could infer that expression of α-tubulin regulated by microenvironmental stiffness in chondrocytes is most likely dependent on the ILK-mediated Rflnb/Smad3 axis, which potentially determines the microtubule assembly and kinetics. In addition, the role of Rflnb in cell morphological remodeling during epithelial-to-mesenchymal transition has been reported to be complicated.^91,92^ Cells overexpressing Rflnb displayed an increase in lamellipodia protrusion at the extended part of the dendritic protrusion,^83^ indicating a role for Rflnb in cell membrane spreading. Therefore, we established an association between cell membrane morphology and microtubule dynamics. Particularly, microtubule dynamics are influenced by the cell cycle.^93^ In particular, we found that microenvironmental stiffness directly regulates chondrocyte microtubule assembly, especially at anaphase, and that stiffened substrates induced higher expression of cyclin B1, cyclin B2, and CDK1 (Fig. S9). Cyclin-dependent kinases (CDKs) are core cell cycle regulators that form complexes with cyclin B to drive the G2-to-mitosis transition.^94,95^ The higher expression of these proteins in chondrocytes on stiff substrates indicates the role of stiffness in accelerating the chondrocyte cell cycle. Collectively, we bridged the clues from cell membrane spreading, microtubule assembly, and cell division regulated by microenvironmental stiffness.

**Fig. 8:**
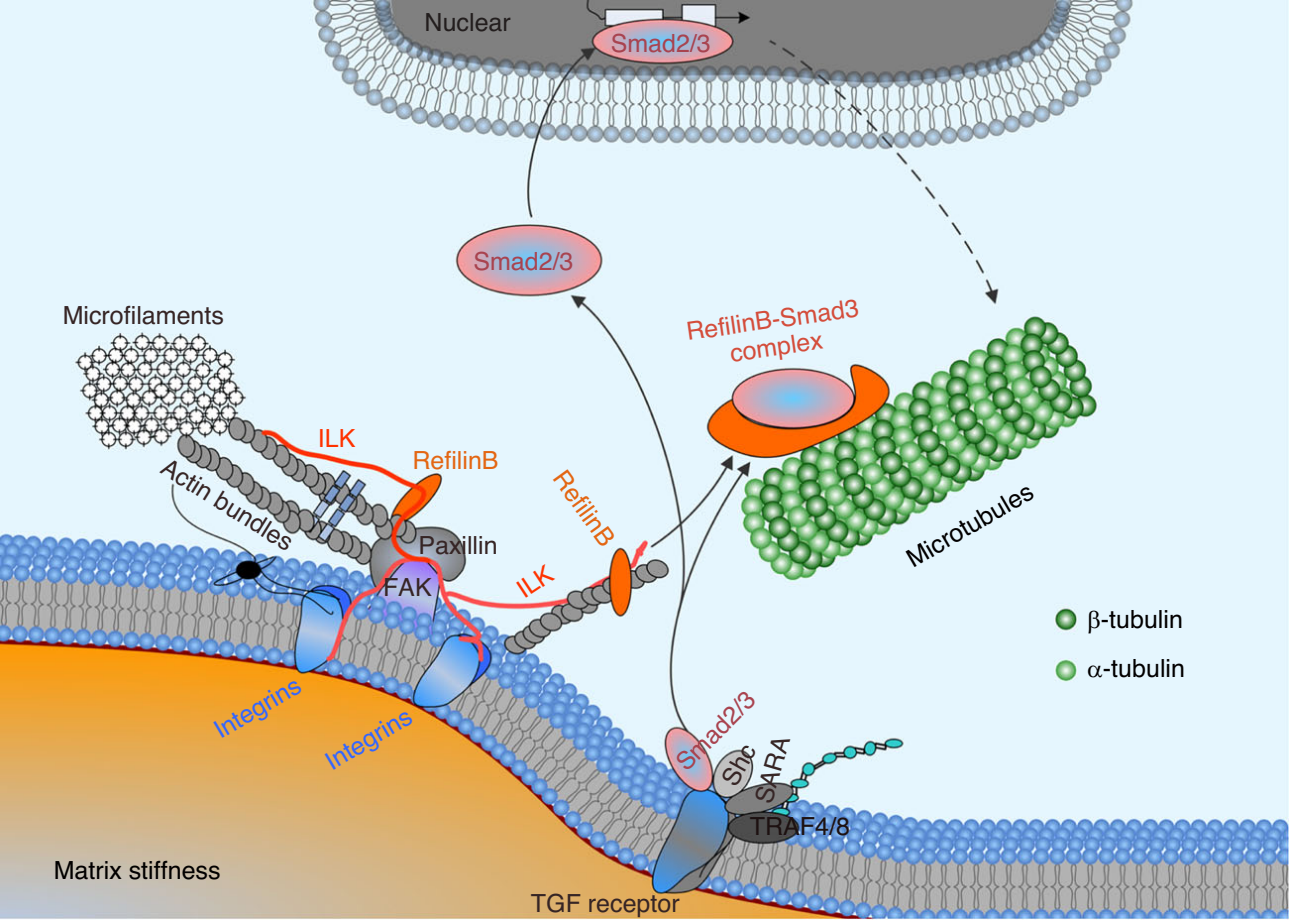
Schematic diagram indicating the role of ILK-refilin B/Smad3 axis in microenvironmental stiffness-mediated microtubule formation. Chondrocytes sense matrix stiffness and respond by altering the expression of interleukin ILK, a mechanically sensitive protein. ILK triggers changes in refilin B and the refilin B–Smad3 complex and regulates microtubule formation

The interaction between mechanical and chemical signals in the microenvironment determines cellular behavior as well as tissue physiology and pathology.^96^ The key step by which cells sense foreign mechanical signals, including ECM stiffness, and integrate them into intracellular chemical signals, such as Smad3 signaling, is of particular importance.^96,97^ Integrins are considered to play an indispensable role in the mechanoperception for shear stress,^98^ static tensile force,^99^ cyclic tensile stress,^100^ hemodynamic force,^101^ static compression^102^ and ECM stiffness.^103,104^ Integrins are a critical type of cell adhesion molecules composed of α and β subunit heterodimers.^105^ To date, 18 α subunits and 8 β subunits have been identified, forming at least 24 distinct integrins.^106^ The mechanosensing subunits primarily include α1/5/6/V and β1/3.^107,108^ Our scRNA-seq results revealed that in chondrocytes, integrins, including Itga4, Itga5, Itga6, Itgal, Itgav, Itgam, Itgb2 and Itgb5, are mechanosensitive to substrate stiffness, with integrin αV showing the pronounced increase on the stiff substrate (Fig. S24). Additionally, pathways associated with mechanochemical transduction and proliferation, including focal adhesion, cell adhesion molecules, actin cytoskeleton regulation, and PI3K-Akt and MAPK signaling, were altered (Fig. 7a). Moreover, changes in the collective signaling pathways highlight the potent role of ILK, a hub for the reception of mechanical signals and initiation of diffusible signals.^53^ We established the interaction between ILK and integrins through protein interaction (Fig. S24b), which confirms the previous results that ILK binds to the intracellular domains of β1/3 subunits to form integrin-ILK complexes and initiate the intracellular mechanotransduction.^109^ In the current study, we detected differential expression of ILK in chondrocytes in response to mechanical stiffness (Fig. 7b, c) and confirmed that it directly mediates the expression of Rflnb and microtubule changes in mitotic chondrocytes (Fig. 7d–h). Notably, ILK and Smad3 signal may have mutual regulatory effects (Fig. 8). On the one hand, ILK, as a hub for mechanical signals, can activate cytoplasmic signals such as the Smad3 signal,^110,111^ and these results were also confirmed by our data (Fig. S26). On the other hand, the activation of Smad3 signaling can induce its nuclear accumulation, which leads to its interaction with transcriptional factors^112^ and thus mediates the transcription of genes, including ILK.^113^ This mutual influence between ILK and Smad3 contributes to an increase in Rflnb levels in chondrocytes cultured on stiffened substrates, thereby promoting the generation of microtubules. This is another regulatory mechanism independent of the direct physical interactions between Rflnb and Smad3.^49^ Based on the above mechanisms, we can interpret the entire process of Rflnb-mediated microtubule formation in chondrocytes in response to mechanical stiffness.

However, we recognize some limitations in the current study. First, noting the fact that more microtubules are formed on stiff substrates, we speculated that microtubule assembly might be directly related to the energy demand of chondrocytes under pathological conditions (stiffened matrix microenvironment). Under pathological conditions, the mode of energy metabolism (whether anaerobic glycolysis or oxidative phosphorylation) in the cartilage may shift.^64,114,115^ As mitochondriopoiesis is closely related to microtubule function, the aerobic phosphorylation of chondrocytes will likely change; however, this needs to be confirmed by future experiments. Second, eight genes (alpha isotypes) encode α-tubulin, and eight genes encode β-tubulin.^116,117^ Although “generic” α-tubulin and β-tubulin isotypes are highly conserved in chondrocyte species, the specific determinants of the subtypes and their mechanism remain to be elucidated. The subtypes of individual changes were identified using RNA sequencing; however, the importance of individual subtypes was not the focus of this study. Third, we believe that the factors regulating microtubule formation induced by substrate stiffness are not limited to Rflnb. RNA sequencing and protein mass spectrometry indicated that a series of factors are involved in the regulation of microtubule morphology. However, the roles of these factors and their underlying mechanisms remain unknown. Nevertheless, this study provides a database for further research. Fourth, to observe changes in cellular microtubules, we mainly focused on cell experiments in vitro and applied siRNAs or inhibitors to investigate the effects of key proteins in the pathway of matrix stiffness-mediated cellular microtubules. When demonstrating the important functions of these key proteins, we cannot provide better methods, such as gene knockout, to validate them, although our experimental results are consistent with existing evidence of gene knockouts, such as Smad3^118^ and ILK.^119^ Finally, this study focused on matrix stiffness-regulated microtubule assembly in vitro; however, the details of microtubule changes were difficult to directly capture in normal and OA cartilage tissues because of the limitations of current experimental techniques and the complexity of cartilage lacunae. Nevertheless, when the matrix stiffness mimicked in vitro approaches the cartilage matrix stiffness in vivo, we can present a more physiological or pathological mechanism of the chondrocyte mechanoresponse, which provides an effective pathway to identify potential mechanobiological therapeutic targets.

## Materials and methods

### Chemical reagents

Unless otherwise stated, all conventional chemical agents used for cell and molecular biology experiments were purchased from Sigma-Aldrich (St. Louis, MO, USA). Materials for chondrocyte culture from Hyclone (Logan, Utah, USA) included high-glucose Dulbecco’s modified Eagle’s medium (DMEM, sh30243.01), fetal bovine serum (FBS, sh30084.03), penicillin-streptomycin solution (sh40003.01), and other reagents used in cell culture such as 1× phosphate-buffered saline (sh30256.01). Antibodies from Abcam (Cambridge, UK) included: Anti-alpha Tubulin (ab52866); Anti-beta Tubulin (ab6046), Cyclin B1 (ab181593), Cyclin B2 (ab185622), and CDK1 (ab201008). The secondary antibodies used were Anti-Mouse IgG H&L (Alexa Fluor 488, ab150113), Anti-Mouse IgG H&L (Alexa Fluor 647, ab150115), Anti-Rabbit IgG H&L (Alexa Fluor 488, ab150073), and Anti-Rabbit IgG H&L (Alexa Fluor 647, ab150075). Antibody against Rflnb (orb183474) was purchased from Biorbyt (Cambridge, UK). Antibodies from Santa Cruz (Delaware Avenue, CA) included: anti-β-actin (sc-47778); and the secondary antibodies included Anti-Mouse IgG (H&L m-IgGκ BP-HRP, sc-516102) and Anti-Rabbit IgG (HRP, sc-2357). Antibodies from Zen Bio (Chengdu, China) included: Anti-alpha Tubulin (R23452); Anti-beta Tubulin (200608); Anti-acetyl-alpha Tubulin (251255) and Anti-GAPDH (R380626). Materials purchased from Beyotime (Shanghai, China) included RIPA lysis buffer (P0013B), western blot primary antibody diluent (P0023A), and a bicinchoninic acid assay kit (BCA, P0010). Polyvinylidene Fluoride PVDF (0000195863) was purchased from Merck Millipore (Billerica, Massachusetts, USA). Super-ECL detection reagent (S8206030) was purchased from Yesen (Shanghai, China). PDMS was purchased from Corning (Sylgard 184, NY, USA). Additionally, for some key proteins such as α- and β-tubulin, we used antibodies from two different companies, which showed to have similar effects in western blotting and immunofluorescence (IF).

### Polydimethylsiloxane (PDMS) substrate preparation

Owing to its good biocompatibility and transparency, PDMS has been widely used as a principal biomaterial to study the interaction between cells and substrate stiffness.^97^ Briefly, soft and stiff PDMS substrates were fabricated by changing the curing component (curing agent, Sylgard184) to an oligomeric base (1:45 and 1:15, v/v). Their physicochemical characteristics have been previously described.^36,97^ Substrates with low stiffness (1:45, ~45 kPa) and high stiffness (1:15, ~450 kPa) that were closer to the ECM stiffness of normal and osteoarthritic cartilage, respectively, were used in this study. Before cell culture, the PDMS substrates were exposed to UV radiation for 1 h for sterilization, and then coated with dopamine (0.12 mg/mL in 1 mg/mL Tris, w/v) for 30 min for hydrophilicity.

### Chondrocyte isolation and culture

All protocols involving mice used in this study were reviewed and approved by the Institutional Review Board (IRB) of West China Hospital of Stomatology (No.WCHSIRB-D-2023-152) before experiments. Mice were obtained from HFK Bioscience (Beijing, China). Primary chondrocytes were isolated using a previously described protocol.^36,64^ Briefly, cartilage was separated from the knee joints of 3-day-old C57BL mice using scissors and tweezers and digested with 0.2% type II collagenase (Sigma) in an incubator at 37 °C overnight. Next, isolated chondrocytes were seeded on 55-cm^2^ cell culture dishes using chondrocyte complete media, containing Dulbecco’s modified Eagle’s medium (high-glucose DMEM, 4 mmol L-glutamine, 0.1 mmol nonessential amino acids, 1% penicillin-streptomycin solution, HyClone, UT) supplemented with 10% (v/v) heat-inactivated FBS with 1% penicillin-streptomycin and cultured in a cell incubator at 37 °C and 5% CO_2_. The medium was replaced every 2 days until the cells reached confluence. The chondrocytes were mainly used at passage 1 (P1).

### Scanning electron microscope (SEM)

To evaluate the morphology of chondrocytes with different substrates stiffness, SEM was performed. Chondrocytes were seeded onto glass slides (15 mm in diameter, 801022; NEST, China) covered with a thin layer of soft and stiff PDMS substrates for 12 or 24 h. After washing three times with 1× PBS, the chondrocytes were fixed with 2.5% glutaraldehyde for 2 h. After the removal of glutaraldehyde, chondrocytes were dehydrated using a gradient (30%, 50%, 70%, 80%, 90%, and 100% alcohol, 15 min per gradient). Finally, the cell samples were coated with a thin gold powder and scanned using a scanning electron microscope.

### Quantitative real-time PCR (qPCR)

Total RNA was extracted from primary articular chondrocytes using the RNeasy Plus Mini Kit (Qiagen, Hilden, Germany), strictly following the manufacturer’s instructions. The extracted RNA samples were quantitatively analyzed using a NanoDrop 2000c spectrophotometer (Thermo Fisher Scientific, USA). Subsequently, the RNA was reverse-transcribed into cDNA using the K1621-RevertAid cDNA Synthesis Kit (Mbi, Lithuania). The reverse transcription products were amplified using an ABI 7500 Real-Time PCR System (Applied Biosystems). GAPDH was used as the internal reference control gene, and the target genes were calculated using the ΔΔCt method (2^-ΔΔCt^). Primer sequences included: GADPH: TGATGGGTGTGAACCACGAG (Forward) and AGTGATGGCATGGACTGTGG (Reverse); α-tubulin: CTGATGTATGCCAAGCGTGC (Forward) and TCCTTCTCTAGGGCAGCCAT (Reverse); β-tubulin: GCTCTTGACCATGAGCGGAA (Forward) and CATATTCACGGCCAGCTTGC (Reverse); Acetyl-alpha tubulin: GCAACCAAGAACGGAAGCAG (Forward) and CTCCCCAGACCCATCCAGTA (Reverse).

### Monolayer scratch wound healing assay

To validate the effects of microenvironmental stiffness and si-Rflnb on chondrocyte migration, chondrocytes were seeded onto soft and stiff substrates. The detailed protocol for the scratch wound-healing assay has been described previously.^68,120^ Notably, a sterile pipette tip was used to gently create a thin linear scratch in the cell monolayer to avoid damaging the surface of the soft/stiff substrates, which could compromise the experimental outcomes. Chondrocyte migration was recorded at 24 and 48 h post-scratch, and migration rates (calculated as migrated cell area/scratched area) were analyzed using the ImageJ software.

### Western blotting

Cells were seeded onto soft and stiff substrates for 72 h, rinsed three times with 1× PBS (ice-cold, PH 7.4), and lysed using cell lysis buffer (RIPA kit) containing phosphatase inhibitors such as orthovanadate (P0013B, Beyotime, Shanghai, China), 2% phenylmethanesulfonyl fluoride (P7626, Sigma), and EDTA-free protease inhibitors. After concentration determination by the BCA assay, 5× loading buffer was added to the protein samples to form a mixture at a 1:1 ratio (v/v). Equal amounts of samples were loaded into the wells of a 10% SDS-PAGE gel, electrophoresed at 120 V for 1.5 h and then transferred to PVDF membranes (1.5 h at 250 mA). Subsequently, the membranes were incubated with relevant primary antibodies, including β-actin (1:1 000, sc-47778), alpha-tubulin (1:1 000, R23452), beta-tubulin (1:1 000, 200608), acetyl-alpha Tubulin (251255), Cyclin B1 (1:1 000, ab181593), Cyclin B2 (1:1 000, ab185622), and CDK1 (1:1 000, ab201008) at 4 °C overnight. After rinsing three times with 1× TBST, the immunolabeled proteins were incubated with the relevant secondary antibodies for 2 h at 20 °C–25 °C. Super Signalling Reagent (Pierce, Rockford, IL, USA) was used to visualize the immunoreactive blots. Finally, the intensity of each protein of interest was normalized to β-actin loading control in the same lane to account for changes.

### Immunofluorescence and CSLM

To investigate microtubule distribution in detail, we analyzed immunofluorescence using CSLM, as previously described.^36,120^ The chondrocytes were cultured on soft and stiff substrates for 72 h. After fixation with 4% paraformaldehyde (PFA) for 5–10 min, the chondrocytes were permeabilized with 0.25% Triton X-100 (Beyotime, Shanghai, China) for 5 min and blocked with 5% BSA (bovine serum albumin (BSA, w/v) for 1 h. Cell samples were incubated with primary antibodies against alpha-tubulin (1:200), beta-tubulin (1:200), acetyl-alpha-tubulin (1:200), and p-Smad3 (1:200) at 4 °C overnight. Then, cells were incubated with secondary antibodies (1:400, AlexaFluor647; Rabbit, ab150075; and 1:400, Alexa Fluor® 647, Mouse, ab150115, respectively) according to the antibody species specificity, for 2 h at RT. The cells were lastly counterstained with 4, 6-diamidino-2-phenylindole (Dapi, 10 μg/mL, D9542, Sigma) for 15 min at RT. Immunofluorescence images were captured using CLSM (FV3000, Olympus, Japan).

### Bulk RNA sequencing

Bulk RNA sequencing and initial data analysis were performed by Shanghai LifeGenes Biotechnology (Shanghai, China). Briefly, after the RNA concentration was determined using the RNA Nano 6000 Assay Kit of the Bioanalyzer 2100 system (Agilent Technologies, CA, USA) and reached the threshold, clustering of the index-coded samples was achieved on a cBot Cluster Generation System using a HiSeq 4000 PE Cluster Kit (Illumina, San Diego, CA, USA). Clean data were acquired from the raw data (fast q-format) by deleting adapter-containing, poly N-containing, and low-quality reads. For data analysis, HTSeq v0.6.1 was used to calculate the number of reads mapped to each gene. Gene data on fragments per kilobase of exon model per million mapped fragments (FPKM) were figured. To test for statistically significant DEGs, KOBAS v3.0. was applied. Biological process analyses based on gene ontology (GO) were performed to determine the mediators involved in the regulation of microtubules during mechanosensation. Kyoto encyclopedia of genes and genomes (KEGG) terms with |FoldChange | ≥1.5 and *P* value < 0.05 was used to distinguish the different expressed genes. The pheatmap and bubble chart were generated using the online R package (R version 4.1.2).

### Single-cell RNA sequencing

Isolated mouse primary chondrocytes were seeded onto stiff and soft substrates for 72 h and collected for scRNA sequencing. Single-cell RNA sequencing (scRNA seq) library was based on the Chromium Single Cell 3ʹ Reagent Kits v3 (10× Genomics). Briefly, after three washes with 0.04% BSA-DPBS, chondrocytes were captured in droplets in a targeted cell recovery solution. Following the step of the reverse transcription, barcoded-cDNA was utilized with 3’ gene expression library construction. Gel beads-in-emulsion (GEMs) were generated by combining Barcoded Single Cell 3ʹv3 Gel Beads. Immediately after GEM generation, the gel beads were dissolved, the primers were released, and the co-partitioned cells were lysed. Primers containing an Illumina TruSeq Read 1, 16 nt 10× Barcode, 12 nt unique molecular identifier and 30 nt poly sequence were mixed with cell lysates. Incubation with GEMs generated barcoded, full-length cDNA from polyadenylated mRNA. TruSeq read 1 was required during GEM incubation. The final library containing primers P5 and P7 was used for Illumina bridge amplification. Finally, the library was sequenced using the NovaSeq platform (Illumina) to generate 150 bp paired-end reads. Owing to the fact that the knee joints of newborn mice are almost entirely composed of chondrocytes and chondrocyte precursor cells that have not yet undergone endochondral ossification, we grouped the cells into chondrocyte subsets including proliferative chondrocytes, prehypertrophic chondrocytes, hypertrophic chondrocytes, and fibrocartilage chondrocytes (FCs) based on the markers described in the previous reports.^121–123^ GO analysis was performed to determine the mediators of mechanosensation involved in the regulation of microtubules. KEGG terms with |FoldChange | ≥1.5 and *P* value < 0.05 were used to distinguish the different expressed genes. The p-heatmap and bubble chart were generated using the online R package, R studio (R version 4.1.2).

### Protein mass spectrometry

After culturing on soft/stiff PDMS substrates for 72 h, chondrocyte samples were lysed into peptide fragments using NP-40 buffer containing a protease and phosphatase inhibitor cocktail (1%, v/v). Next, the peptide samples were cleaned by removing contaminants with trichloroacetic acid/acetone, solubilized in 0.2% RapiGest in ammonium bicarbonate, and the peptide extracts were trypsinized in a protease solution. Subsequently, the samples were loaded onto an LTQ-Orbitrap instrument. The tryptic peptide mixtures were auto-sampled onto a 15-cm-long 75-μm inside-diameter column and eluted with a linear gradient from 8% to 40% MeCN for 2 h. Lastly the separated peptides were electrosprayed into the LTQ-Orbitrap mass spectrometer, and then the tandem mass spectra were acquired. GO analysis was performed to identify the mediators involved in the regulation of microtubule mechanosensation. KEGG terms with |FoldChange | ≥1.5 and *P* value < 0.05 were used to distinguish the different expressed proteins. A preheat map was generated using the online R package (R version 4.1.2).

### si-RNA interference

Small interfering RNA (siRNA) was used to confirm the influence of Rflnb and ILK on microtubule formation. The siRNA plasmid was purchased from Santa Cruz Biotechnology (sc-145012, sc-35666) and cell transfection was performed using Lipofectamine RNAiMAX (Invitrogen, Burlington, ON, Canada). Transfection was performed according to the manufacturer’s instructions. The siRNA control group was designated as the scramble group.

### Statistical analysis

All experiments and analyses were repeated at least in triplicate (*n* ≥ 3). All data are presented as mean ± standard deviation (SD). Statistical differences were analyzed using a two-tailed Student’s *t* test to evaluate the variability between two sets, and one-way analysis of variance was used to evaluate the variability between multiple sets. Statistical data analyses are shown in Supplementary Source Data. Post-hoc analysis employed Fisher’s protected least significant difference (PLSD), and the threshold of significance levels was set at 0.05.

## Supplementary information

SUPPLEMENTAL MATERIAL

## Source data

Source Data

## Data Availability

Any data used in this study are available from the corresponding authors upon request, in addition to the attached Supplementary materials.
