# Supplementary material for: Microenvironmental stiffness directs microtubule perturbation in chondrocyte mitosis via ILK-refilinB/Smad3 axis
Source: Bone Res. 2026 Jan 30;14:15. doi: 10.1038/s41413-025-00491-4 (PMC12858872; doi:10.1038/s41413-025-00491-4)
Supplement: Supplementary file 1 — SUPPLEMENTAL MATERIAL [file 41413_2025_491_MOESM1_ESM.doc]

Supplementary materials for *Original paper*

**Microenvironmental stiffness directs microtubule perturbation in chondrocyte mitosis via ILK-refilinB/Smad3 axis**

Mengmeng Duan, Chenchen Zhou, Guanyue Su, Chunhe Zhang, Jie Ren, Qingjia Chi, Xiaojing Liu, Li Yang, Haiqing Bai, Yang Claire Zeng, Seongmin Kim, Yunhao Zhai, Crystal Yuri Oh, Adam Yongxin Ye, Yuting Chen, Longlong Si, Xiaoheng Liu, Jing Xie

**Supplementary figures**

**Figure S1**

**
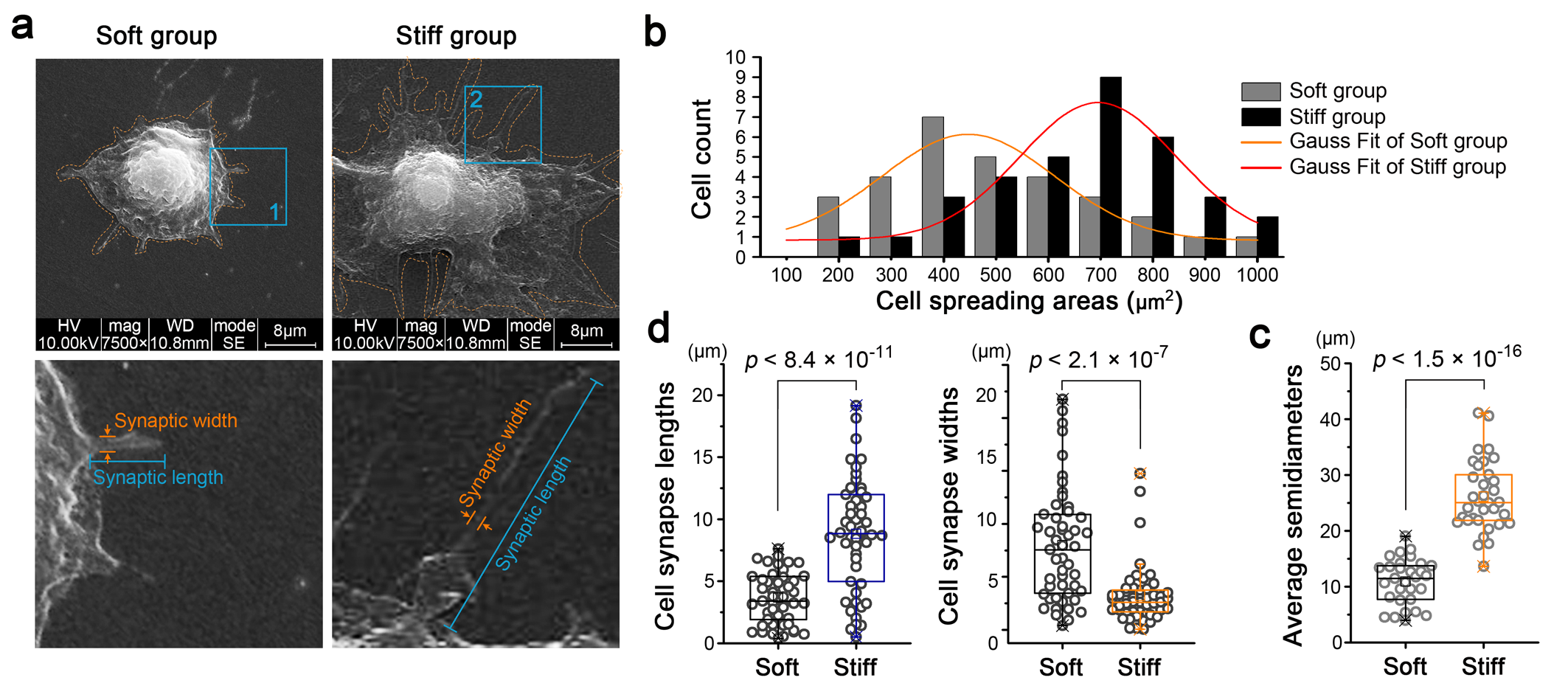
**

**Figure S1. Cell morphological changes of chondrocytes in response to the soft/stiff substrates (**This supplementary figure is related to Figure 1a**).**

**a.** SEM images showing the changes in cell spreading areas (yellow dashed curves), synaptic lengths (cyan), and synaptic widths (yellow arrows).

**b.** Quantitative analysis indicating the changes of cell spreading areas in chondrocytes seeded onto the soft/stiff substrates. The quantification was based on total 30 (soft) and 34 (stiff) cells, respectively from three independent experiments. The fitting curves are based on Gaussian curve implementation.

**c.** Quantitative analysis showing the changes in average cellular semidiameter in chondrocytes seeded onto the soft/stiff substrates. All significance data presented are based on two-tailed Student’s t tests.

**d.** Quantitative analysis showing the changes in synaptic lengths (left) and widths (right) in chondrocytes seeded onto the soft/stiff substrates. All significance data presented are based on two-tailed Student’s t tests.

**Figure S2**


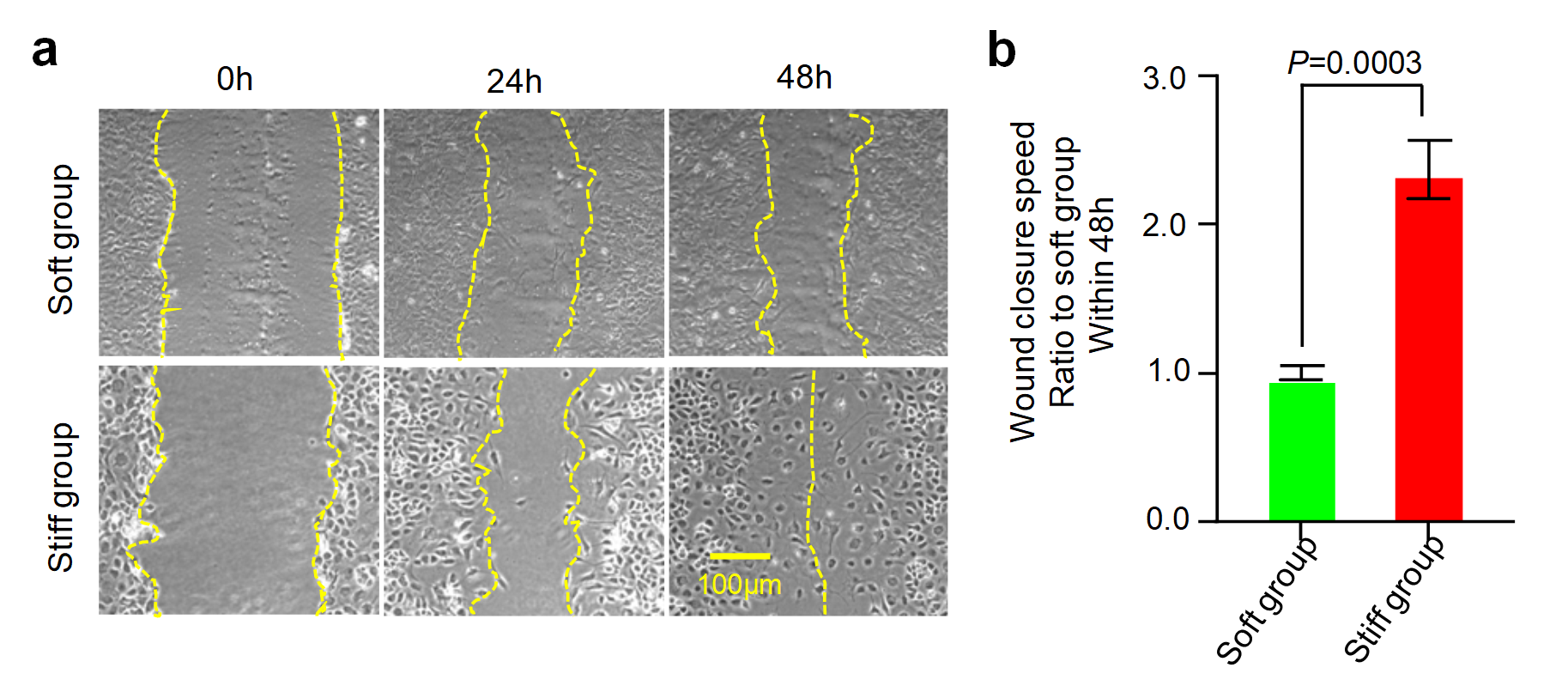


**Figure S2. Cell migration changes in chondrocytes seeded onto the soft/stiff substrates for 48 h.**

**a.** The scratch assay showing the change in cell migration of chondrocytes seeded on the soft/stiff substrates for 48 h. The images are chosen based on three independent experiments (n = 3).

**b.** Quantitative analysis confirming the changes in chondrocyte migration seeded on the soft/stiff substrates. Data are based on three independent experiments (n = 3).

**Figure S3**


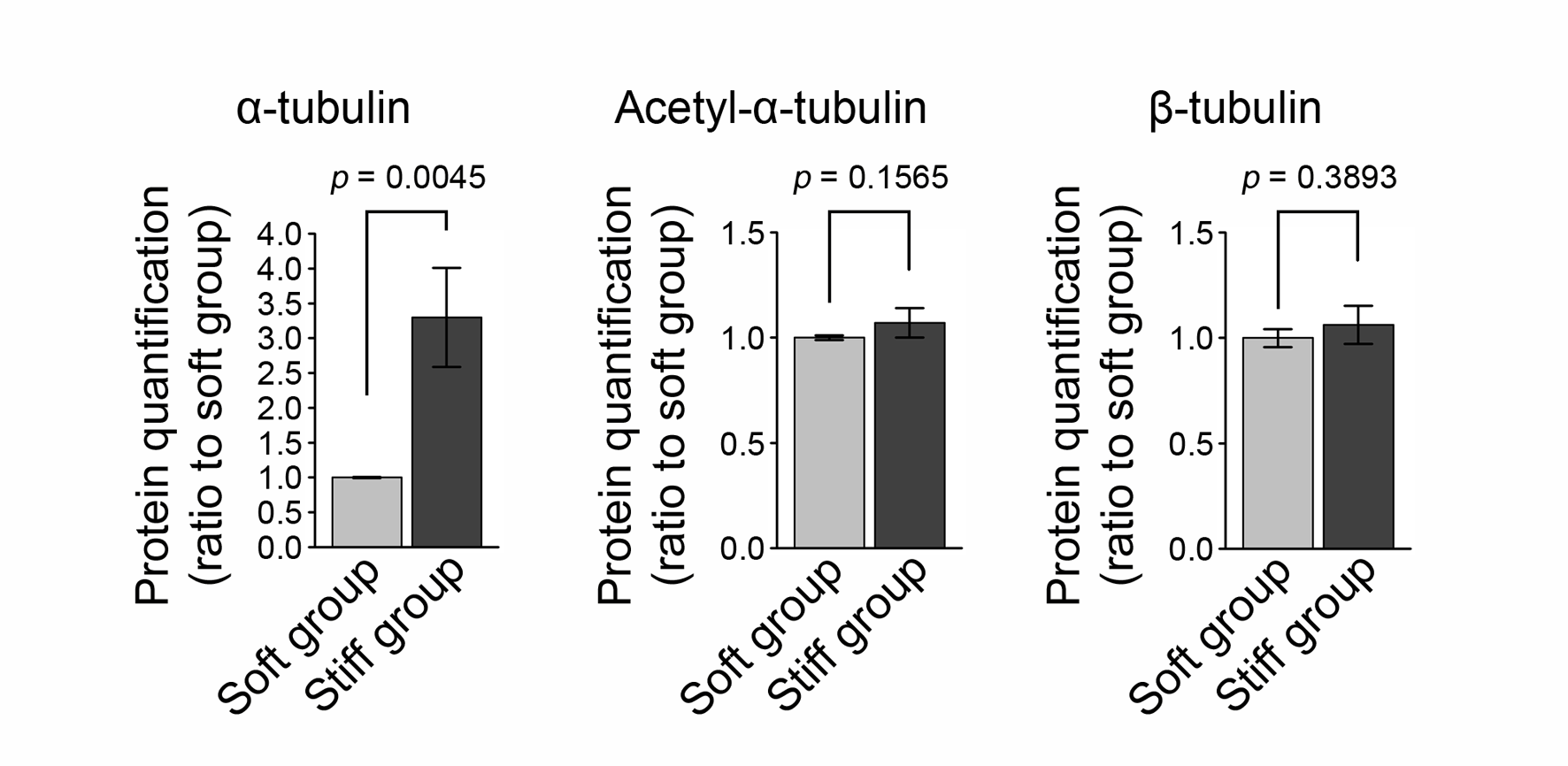


**Figure S3.** Quantitative analysis of α-tubulin, acetyl-α-tubulin and β-tubulin in chondrocytes in response to the soft/stiff substrates. GAPDH and β-actin were used as internal controls (n = 3). The quantifications of target proteins are calculated ratio to β-actin. All significance data presented are based on two-tailed Student’s t tests. This supplementary figure is related to Figure 1e.

**Figure S4**


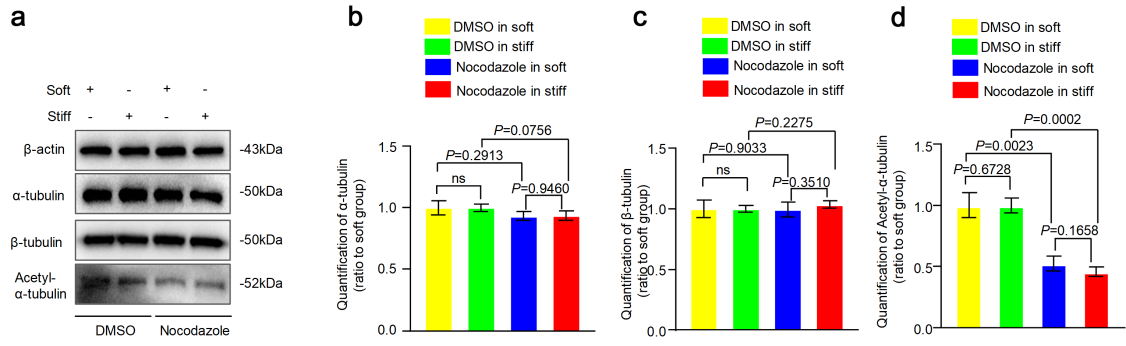


**Figure S4. The expression of tubulins in chondrocytes seeded onto the soft/stiff substrates after treatment with nocodazole for 4 h.**

**a.** Western blotting showing the expressions of tubulins in chondrocytes treated with nocodazole for 4 h. Images are chosen based on three independent experiments (n = 3).

**b-d.** Quantitative analysis showing the expression changes of tubulins in chondrocytes treated with nocodazole. The quantifications of target proteins are calculated ratio to β-actin. Data are based on three independent experiments (n = 3). Significance data presented are based on two-tailed Student’s t tests.

**Figure S5**

**
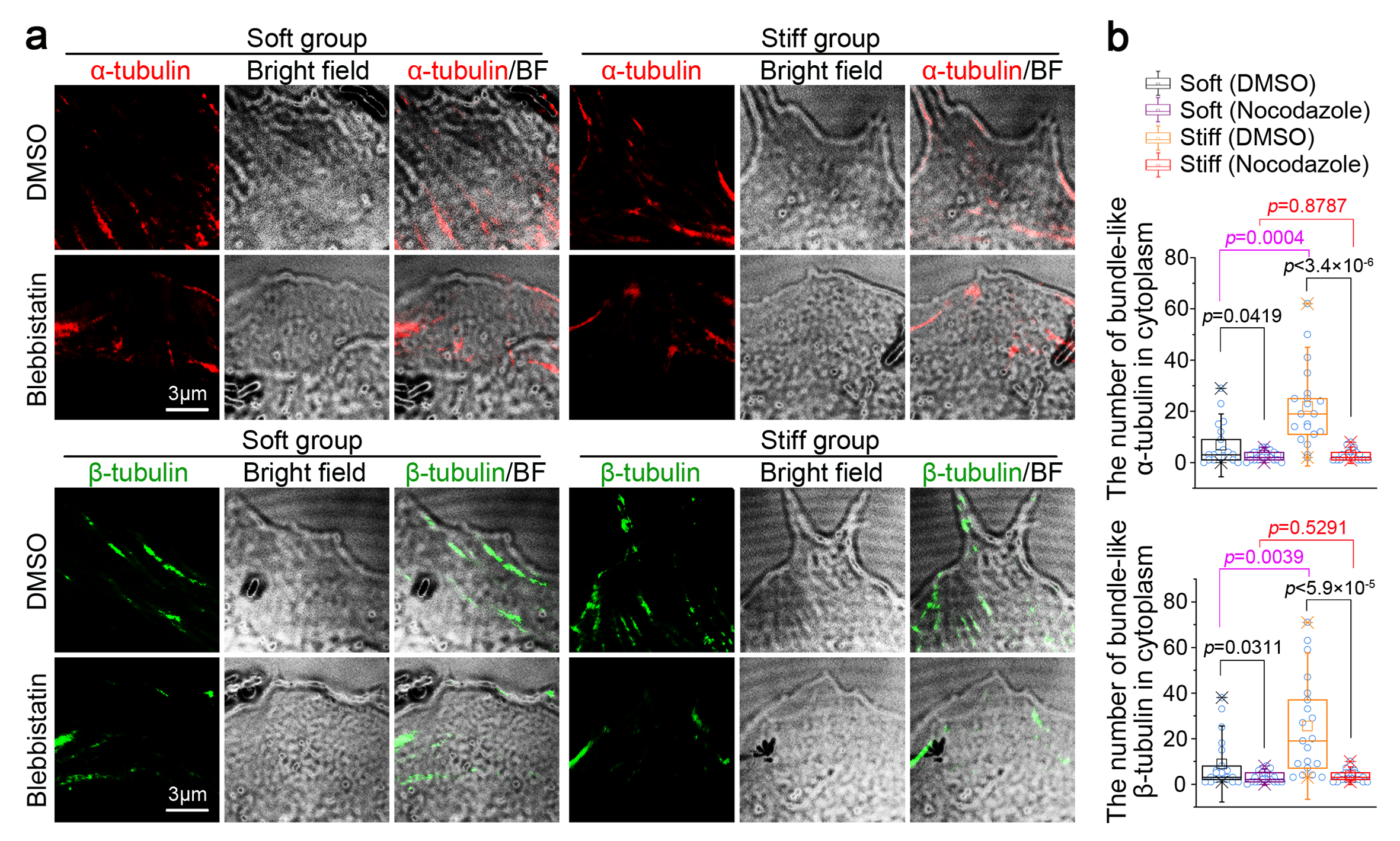
**

**Figure S5. Blebbistatin disturbs distributions of α- and β- tubulin in the peripheral cytoplasmic region of chondrocytes.**

**a.** Representative CLSM images showing that blebbistatin disturbs the distribution of α-tubulin (upper, red) and β-tubulin (lower, green) in chondrocytes. Bright field indicates the cellular synaptic morphology at the cell membrane edge (n = 5).

**b.** Quantitative analysis indicating number changes of bundle-like α-/β-tubulin in the peripheral cytoplasmic region. The results are based on 20 cells from 5 independent experiments.

**Figure S6**


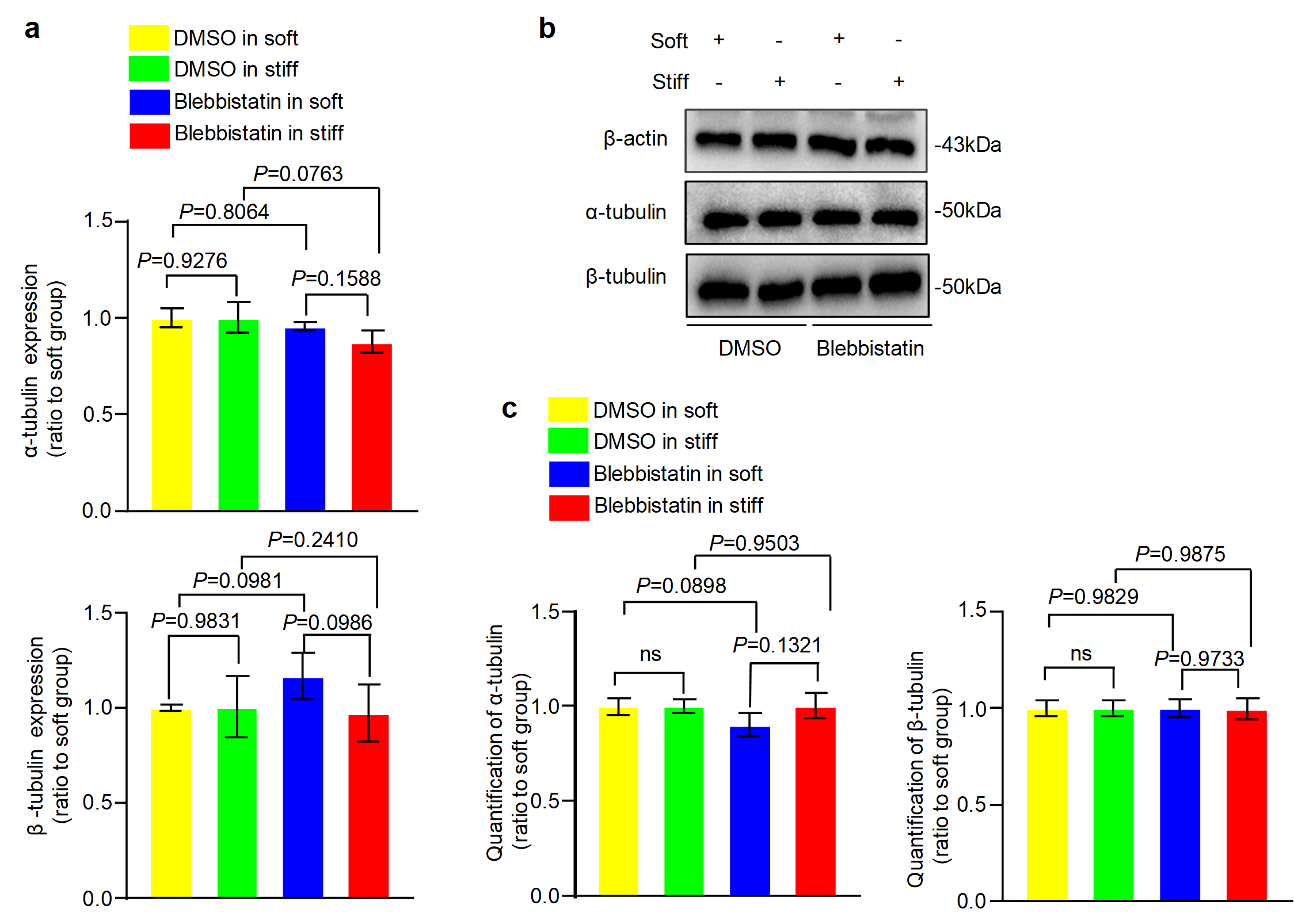


**Figure S6. The influence of blebbistatin on the expression of tubulins in chondrocytes in response to the soft /stiff substrates.**

**a.** qPCR showing that the effects of blebbistatin on gene expression of α-tubulin and β-tubulin in chondrocytes seeded on the soft /stiff substrates. Data are based on three independent experiments (n = 3).

**b.** Western blotting showing that the effects of blebbistatin on the expression of α-tubulin and β-tubulin in chondrocytes seeded on the soft /stiff substrates. Images are chosen based on three independent experiments (n = 3).

**c.** Quantitative analysis confirming the fold change of α- and β-tubulin proteins in chondrocytes treated with blebbistatin in (b). The quantifications of target proteins are calculated ratio to β-actin. Data are based on three independent experiments (n = 3). Significance data presented are based on two-tailed Student’s t tests.

**Figure S7**


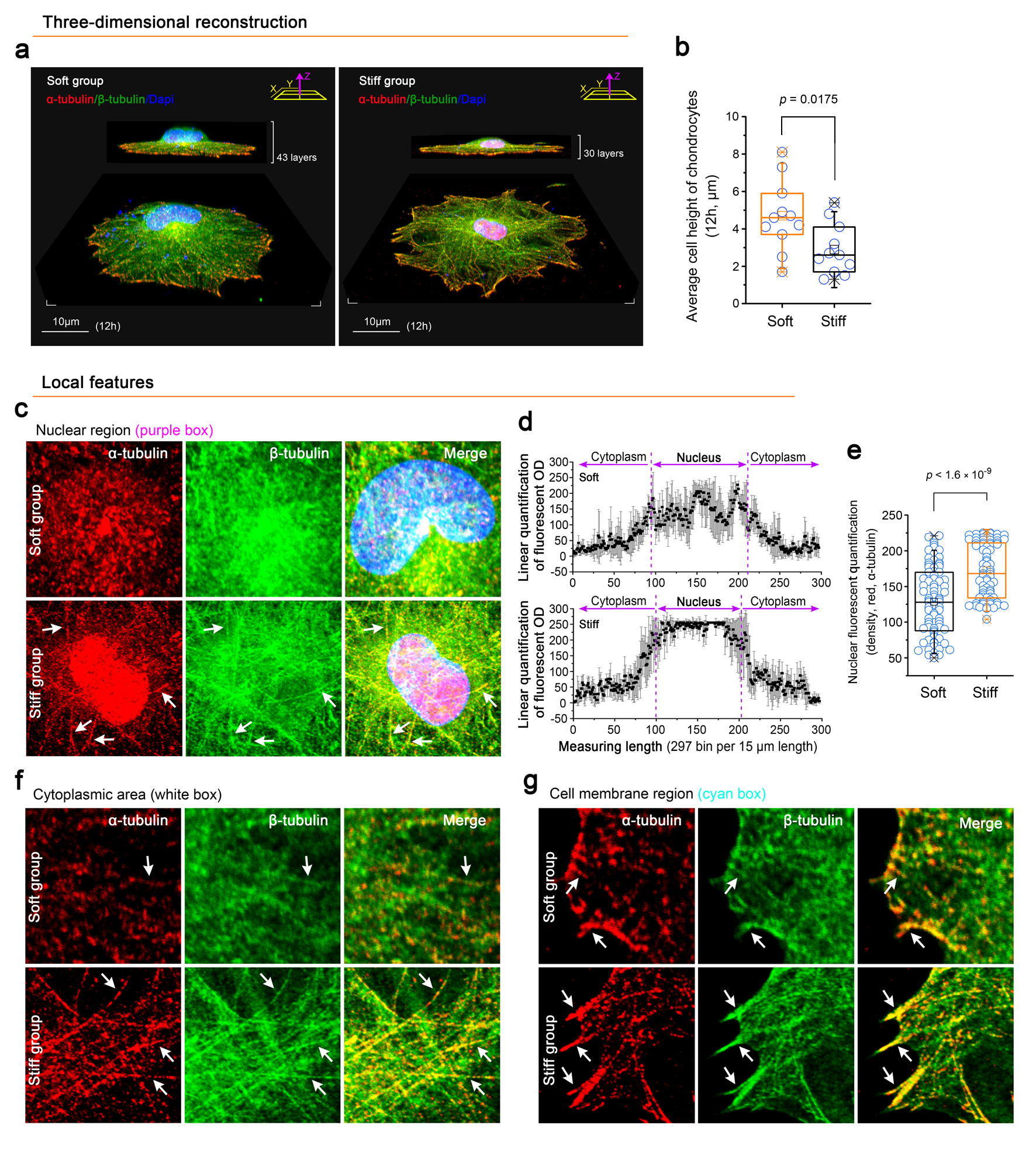


**Figure S7. Details of intracellular microtube distributions in chondrocytes in response to the soft/stiff substrates.**

**a.** Confocal 3D imaging showing the changes of microtubules in individual chondrocytes in response to soft/stiff substrates. Confocal layer-scanning was applied to obtain 3-D reconstructed images of microtubes. Scanning layers are determined by actual cell height. This supplementary figure is related to Figure 3b.

**b.** Box & whisker plot showing the cell height change of chondrocytes in response to soft/stiff substrates. The result is based on 11 cells from 3 independent experiments.

**c.** CLSM image showing the distribution of microtubules at nuclear regions of chondrocytes in response to soft/stiff substrates. This supplementary figure is related to Figure 3b-purple boxed areas. White arrows indicate the microtubules in nuclear region.

**d.** Linear quantitative analysis of immunofluorescence indicating the concentration of α-tubulin in and around nuclear regions.

**e.** Quantitative analysis of total OD intensity in the nuclear region showed that the expression of α-tubulin (red) was much higher in chondrocytes in the stiff group than those in the soft group. The result is based on 80 cells from 6 independent experiments (Source data).

**f.** CLSM image showing the distribution of microtubules at cytoplasmic regions of chondrocytes in response to soft/stiff substrates. This supplementary figure is related to Figure 3b-white boxed areas. White arrows indicate the microtubules in the cytoplasm.

**g.** CLSM image showing the distribution of microtubules at cell membrane boundary of chondrocytes in response to soft/stiff substrates. This supplementary figure is related to Figure 3b-cyan boxed areas. White arrows indicate the microtubules at the boundary of the cell membrane.

**Figure S8**


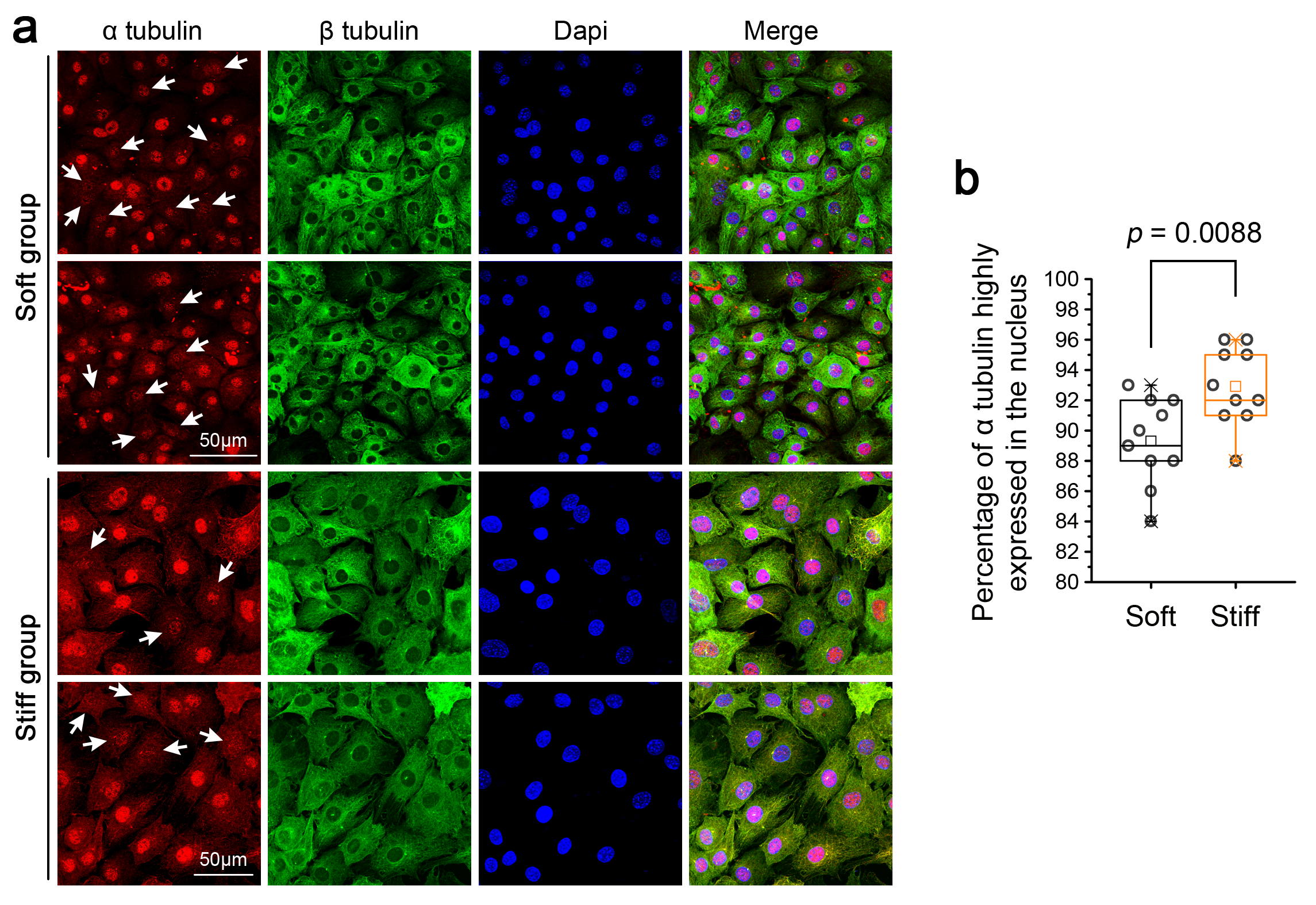


**Figure S8. Different aggregation of α-tubulin in the nuclei of chondrocytes seeded onto the soft/stiff substrates.**

**a.** CLSM images showing the nuclear aggregation of α-tubulin did not fully appear in all chondrocytes seeded onto the soft/stiff substrates. The images are obtained from three independent experiments (n = 3). White arrows indicate the cells that do not aggregate α-tubulin in the nuclear regions.

**b.** Quantitative analysis confirming the percentage of chondrocyes that aggregate α-tubulin in the nuclear region in response to the soft/stiff substrates. Data are based on three independent experiments (n = 3).

**Figure S9**

**
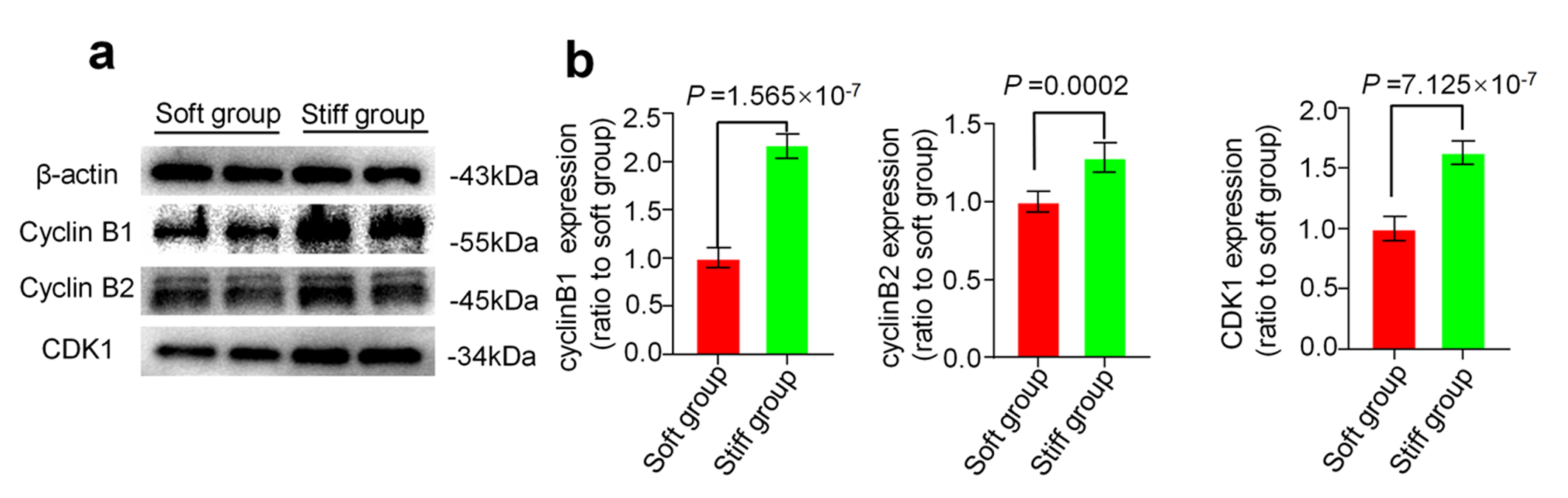
**

**Figure S9. The expressions of cell cycle-related proteins in chondrocytes seeded onto the soft/stiff substrates.**

**a.** Western blotting showing the expression of cyclin B1, cyclin B2 and CDK1 in chondrocytes seeded on the soft/stiff substrates. Data are based on three independent experiments (n = 3).

**b.** Quantitative analysis confirming the fold change in cyclin B1, cyclin B2 and CDK1 proteins in chondrocytes seeded on the soft/stiff substrates. The quantifications of target proteins are calculated ratio to β-actin. The data are based on three independent experiments (n = 3).

**Figure S10**


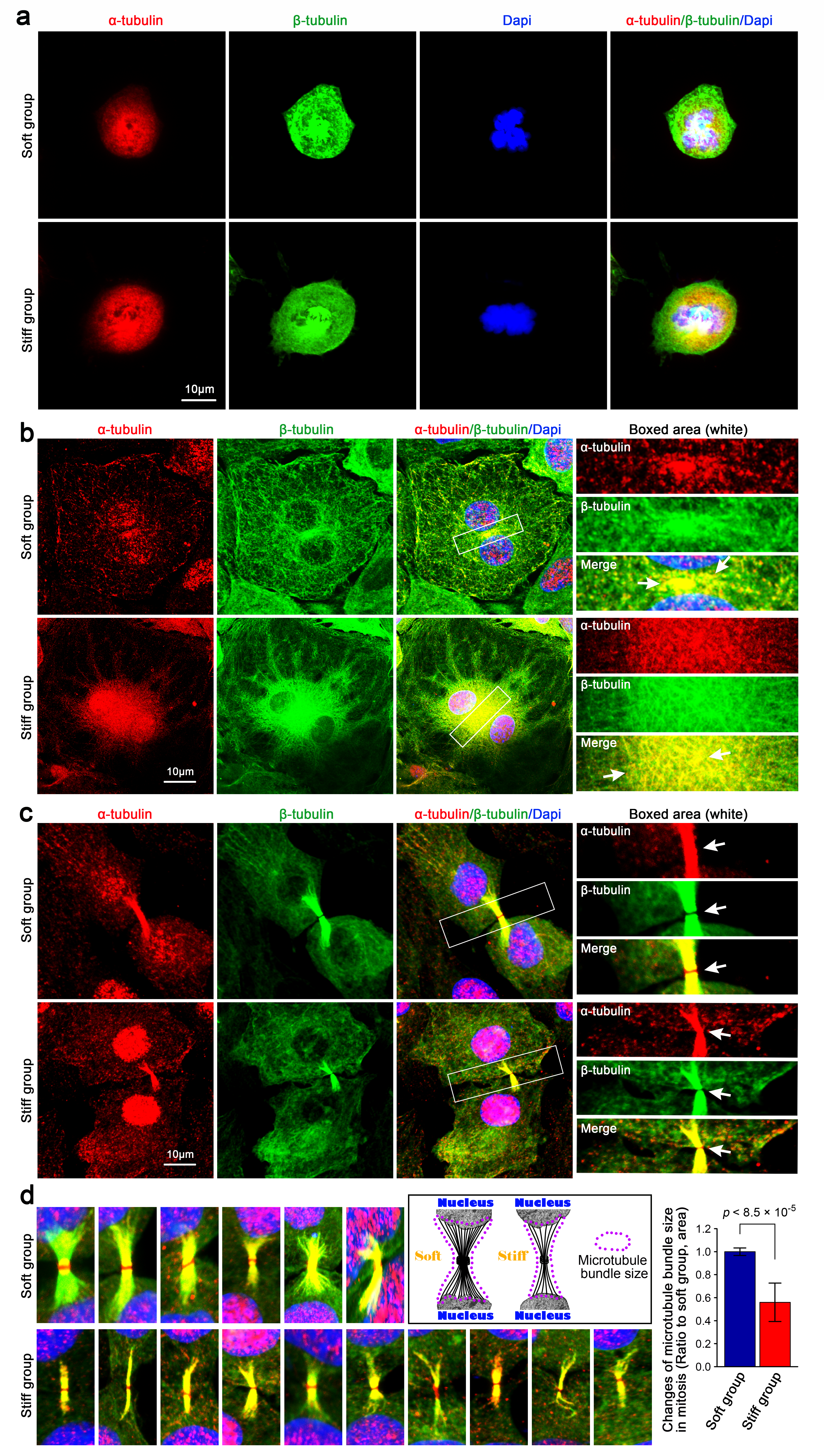


**Figure S10. Distribution difference of microtubules in mitotic chondrocytes (This supplementary figure is related to Figure 3d).**

**a.** Representative CLSM images showing the distribution of microtubules in chondrocytes in response to the soft/stiff substrates at prometaphase (n = 3).

**b.** Representative CLSM images showing the distribution of microtubules in chondrocytes in response to the soft/stiff substrates between anaphase and telophase when round matured offspring nuclei form but cytoplasmic separation has not yet occurred (n = 5). Boxed areas (right) indicate the difference in microtubules at the middle site between two intact nuclei (white arrows indicate).

**c.** Representative CLSM images showing the distribution of microtubules in chondrocytes in response to the soft/stiff substrates after telophase when the microtubules connecting the two progeny cells were segmented and finally disrupted (n = 5). Boxed areas (right) indicate morphological differences in microtubule nodes connecting the two independent progeny cells. White arrows indicate the rupture of microtubule connections between two cells.

**d.** CLSM images showing morphological differences in microtubule nodes between two independent progeny cells after telophase in response to soft/stiff substrates (left), and quantitative analysis indicating the size changes in microtubule nodes between two independent progeny cells at the late stage of mitosis in response to soft/stiff substrates (right). Significance data presented are based on two-tailed Student’s t tests.

**Figure S11**


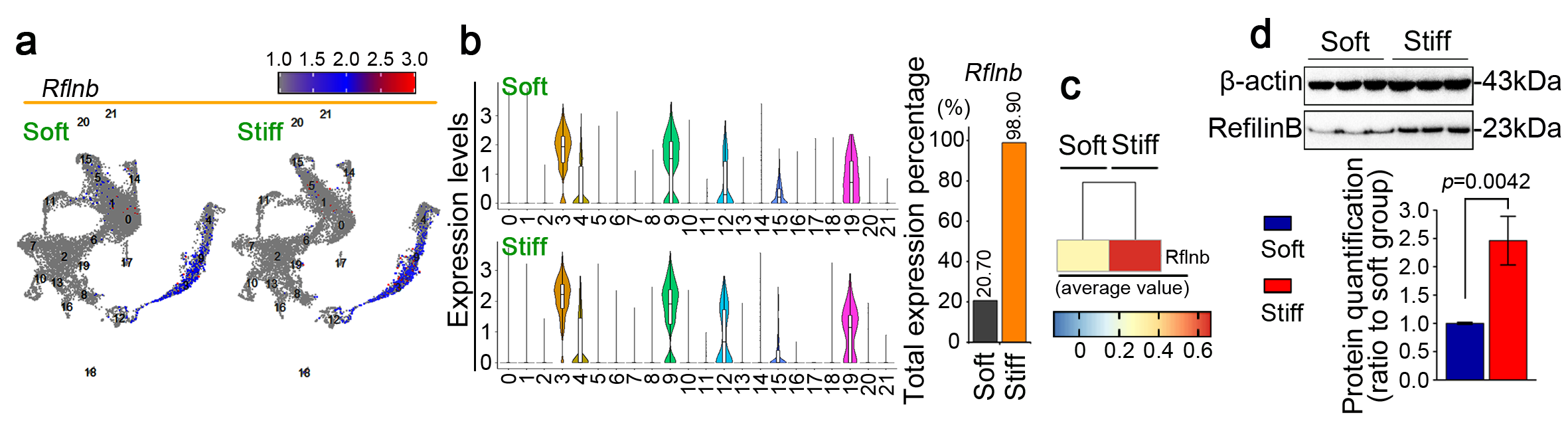


**Figure S11. Expression of refilinB by single-cell RNA-seq and western blotting.**

**a.** Umap based single-cell RNA-seq showing the expression distribution of refilinB (Rflnb) in 21 cell subsets.

**b.** Expression levels of Rflnb in 21 cell subsets in response to soft/stiff substrates.

**c.** Pheatmap showing the average gene expressions of Rflnb by single-cell RNA-seq in chondrocytes in response to soft/stiff substrates.

**d.** Western blotting showing the changes of refilinB protein in chondrocytes in response to soft/stiff substrates (upper) and quantitative analysis confirming the protein changes (lower). β-actin were used as internal controls (n = 3). Quantitative analysis of refilinB is calculated ratio to β-actin.

**Figure S12**


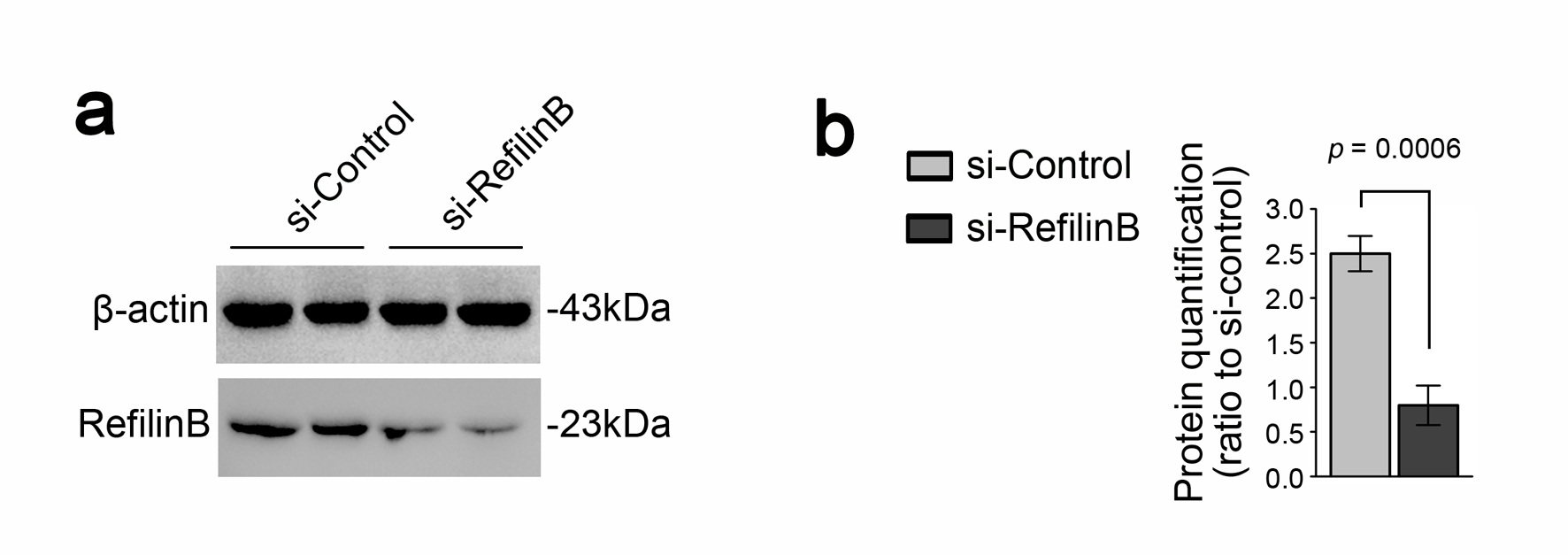


**Figure S12. The knockdown efficiency of refilinB in chondrocytes cultured onto the normal Petri dish by si-refilinB.**

**a.** Western blotting showing the protein changes of refilinB in chondrocytes by siRNA. Images are chosen based on three independent experiments (n = 3).

**b.** Quantitative analysis showing the knockdown efficiency of refilinB in in chondrocytes by siRNA. Quantification of refilinB is calculated ratio to β-actin. Data are based on three independent experiments (n = 3). Significance data presented are based on two-tailed Student’s t tests. This supplementary figure is related to Figure 5a.

**Figure S13**


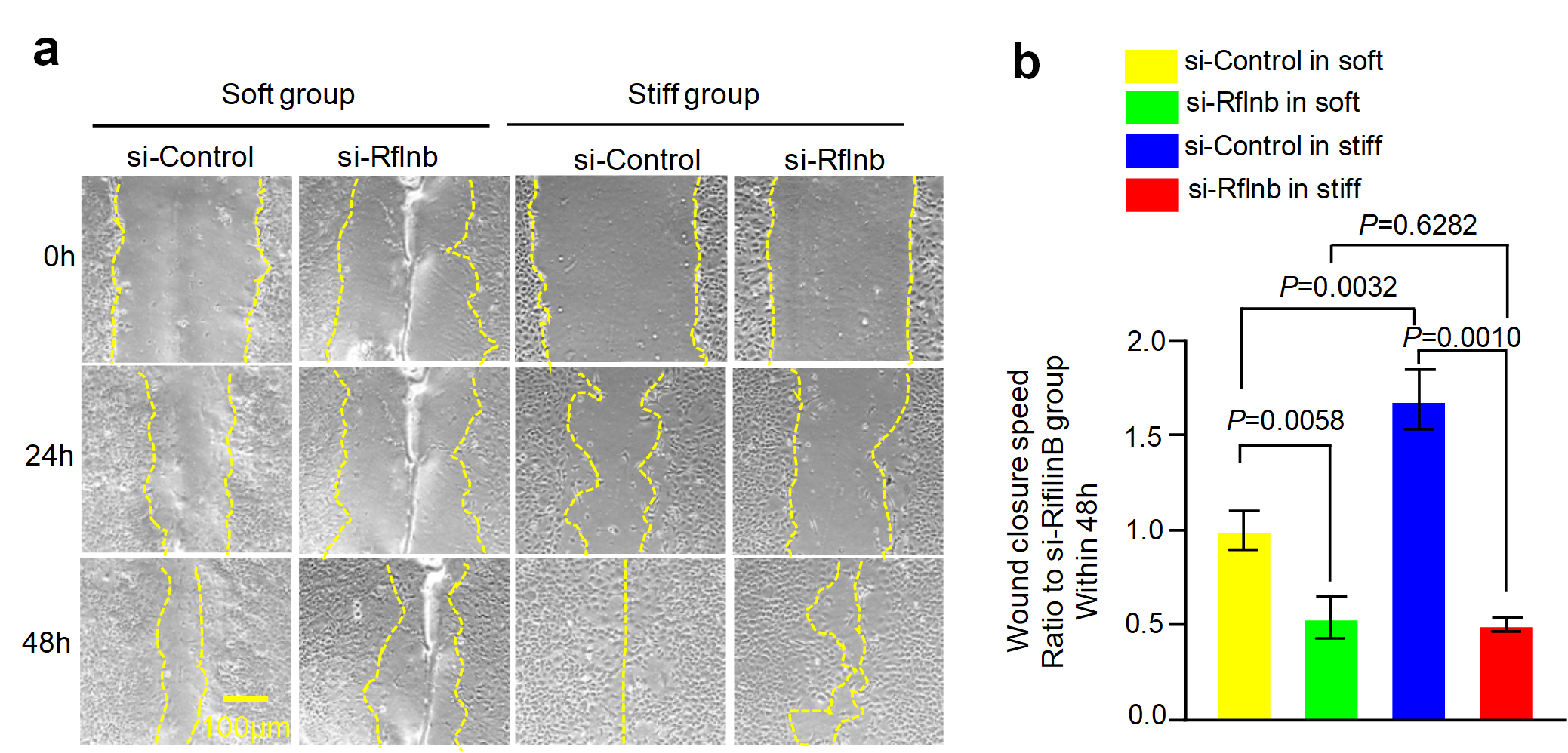


**Figure S13. Cell migration changes in chondrocytes induced by si-refilinB seeded onto the soft/stiff substrates for 48 h.**

**a.** The scratch assay showing the changes in cell migration of chondrocytes induced by si-refilinB seeded on the soft and stiff substrates for 48 h. The images are chosen based on three independent experiments (n = 3).

**b.** Quantitative analysis confirming the changes in chondrocyte migration induced by si-refilinB seeded on the soft and stiff substrates. Data are based on three independent experiments (n = 3).

**Figure S14**


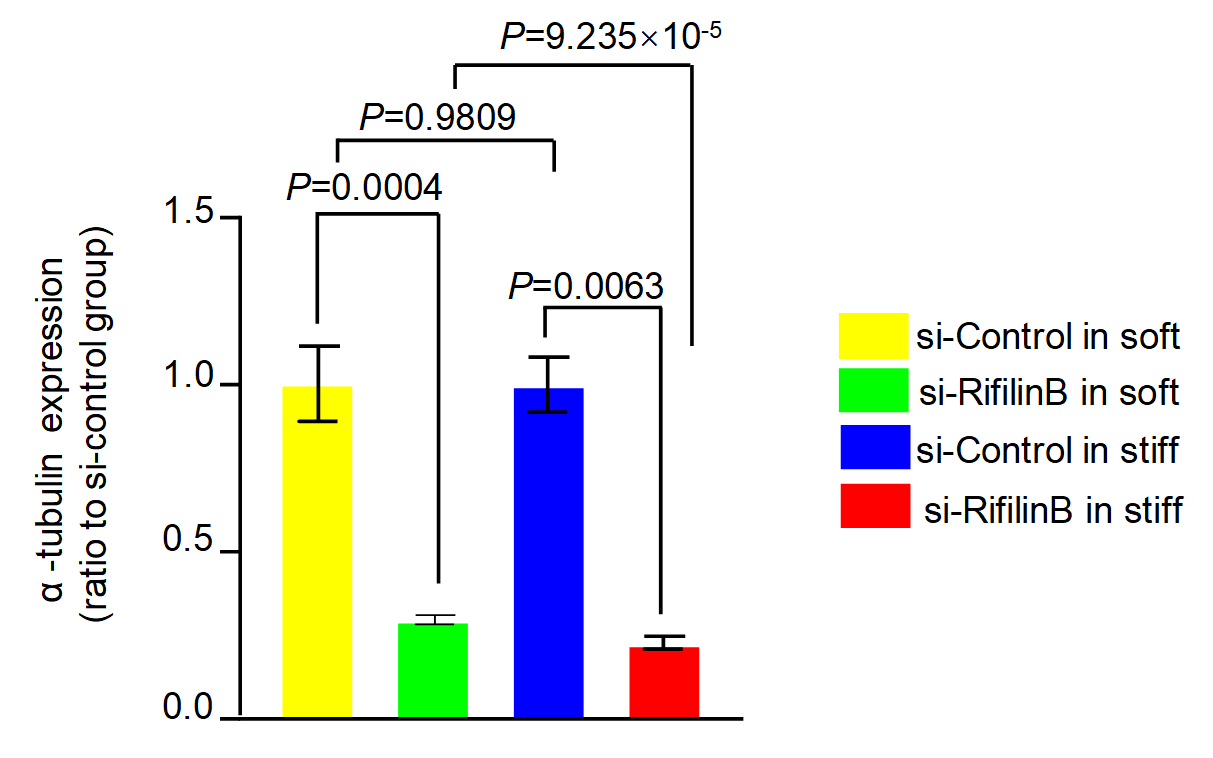


**Figure S14. Gene expression of α-tubulin in chondrocytes on the soft/stiff substrates induced by si-refilinB.** Data are based on three independent experiments (n = 3). Significance data presented are based on two-tailed Student’s t tests.

**Figure S15**


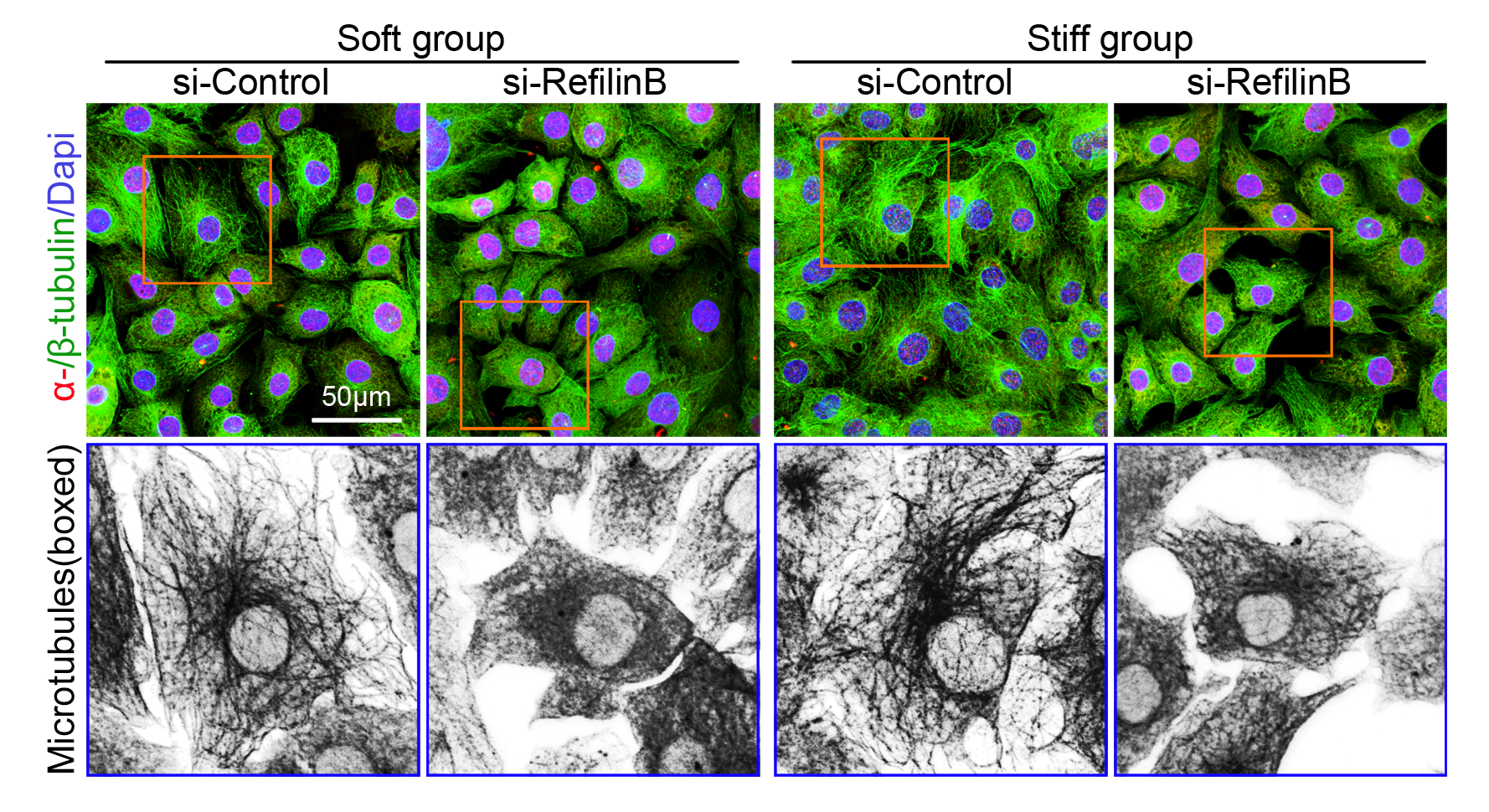


**Figure S15.** Representative CLSM images showing the microtubule changes in a collective of chondrocytes on the soft/stiff substrates by si-refilinB. The boxed areas indicate microtubule changes of chondrocytes (n = 3).

**Figure S16**


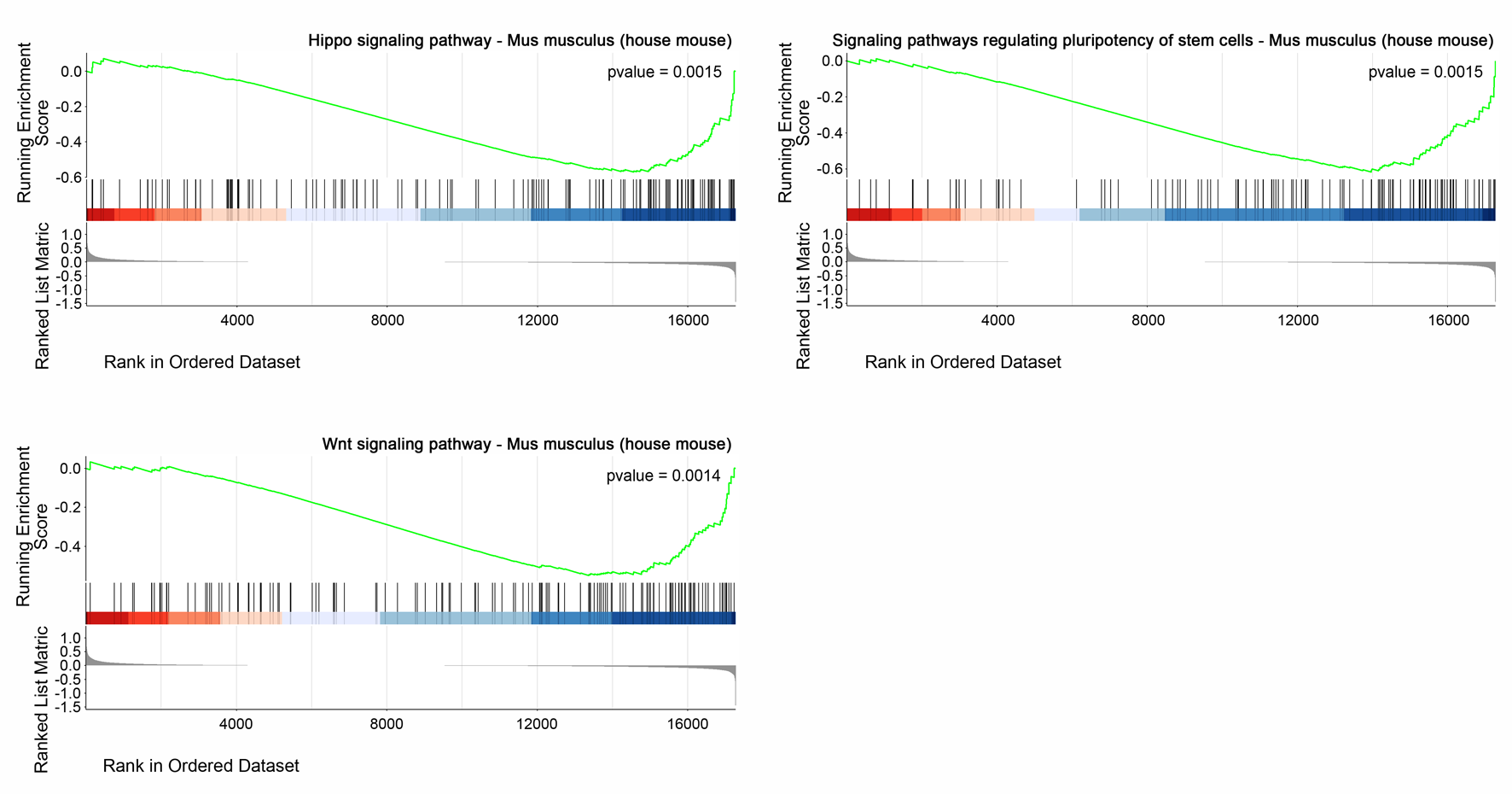


**Figure S16.** Enrichment plots from gene set enrichment analysis (GSEA) showing the signaling pathways, including hippo signaling, signaling pathways regulating pluripotency of stem cells and wnt signaling, which all correlates with the changes of smad3 (source data), in chondrocytes in response to substrates with different stiffnesses by single-cell RNA sequencing.

**Figure S17**


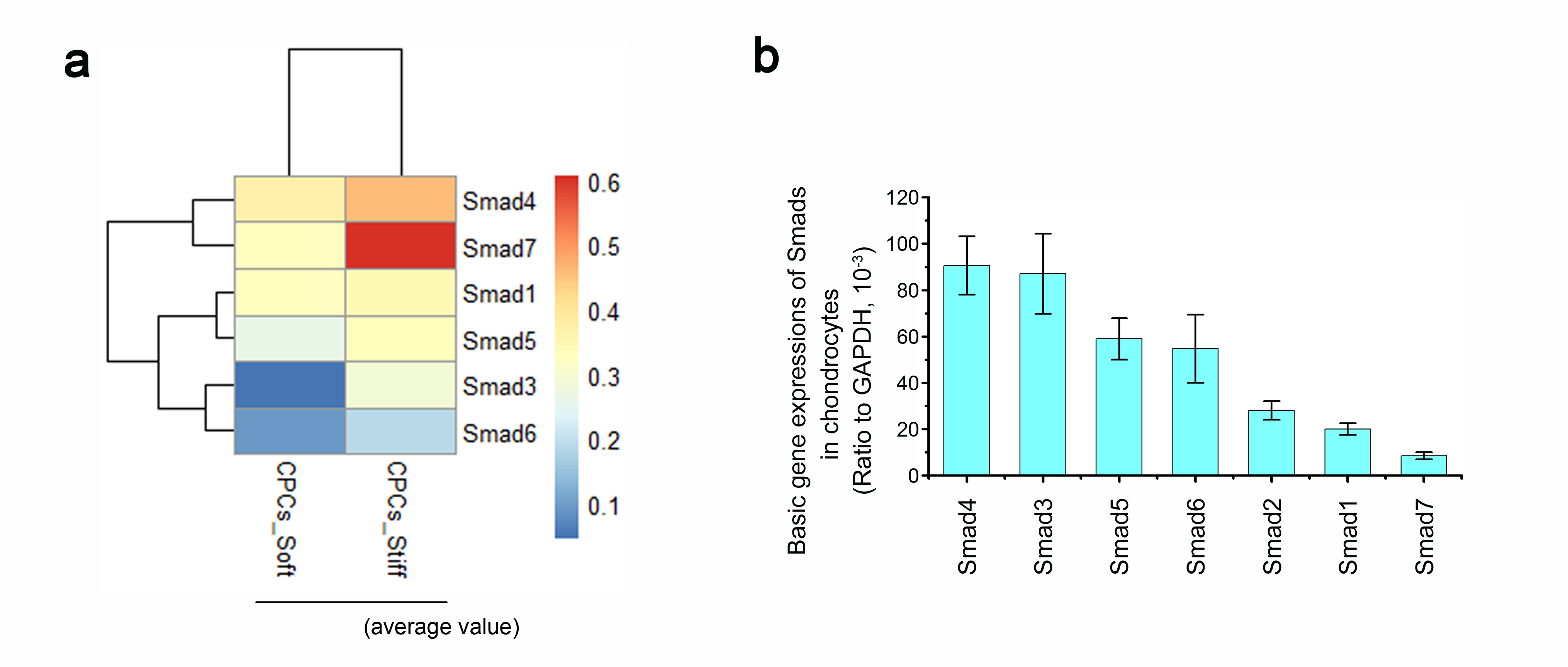


**Figure S17. Different expression of smads in chondrocytes in response to the soft/stiff substrates.**

**a.** Pheatmap showing the changes of Smads in chondrocytes in response to the soft/stiff substrates based on scRNA-seq. The data were presented as average values of expressions. These data are all based on p < 0.05.

**b.** The basal expression of smads in chondrocytes based on scRNA-seq. The data are presented as average values of expressions (n = 3).

**Figure S18**


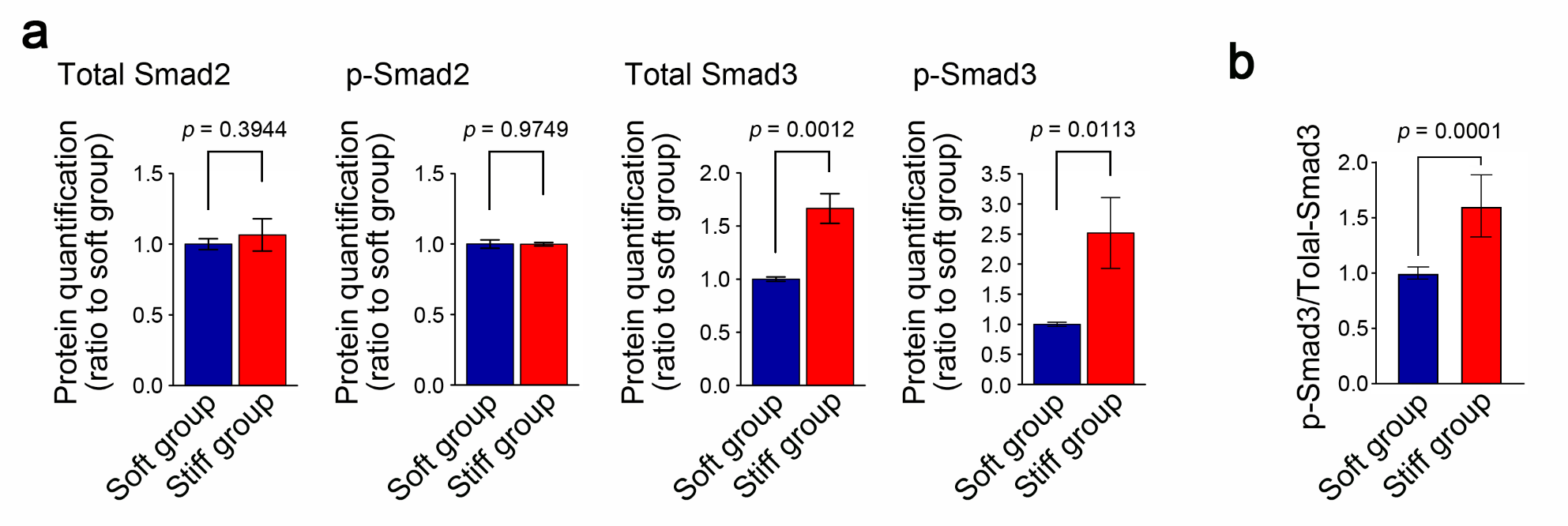


**Figure S18. Quantitative analysis of smad2 and smad3** (This supplementary figure is related to Figure 6b).

**a.** Quantitative analysis of total smad2, phosphorylated smad2, total smad3 and phosphorylated smad3 in chondrocytes in response to the soft/stiff substrates. β-actin were used as internal controls (n = 3). The quantifications of target proteins are calculated ratio to β-actin. All significance data presented are based on two-tailed Student’s t tests.

**b.** The expression ratio of p-Smad3 to total Smad3. Data are based on three independent experiments (n = 5). Significance data presented are based on two-tailed Student’s t tests.

**Figure S19**


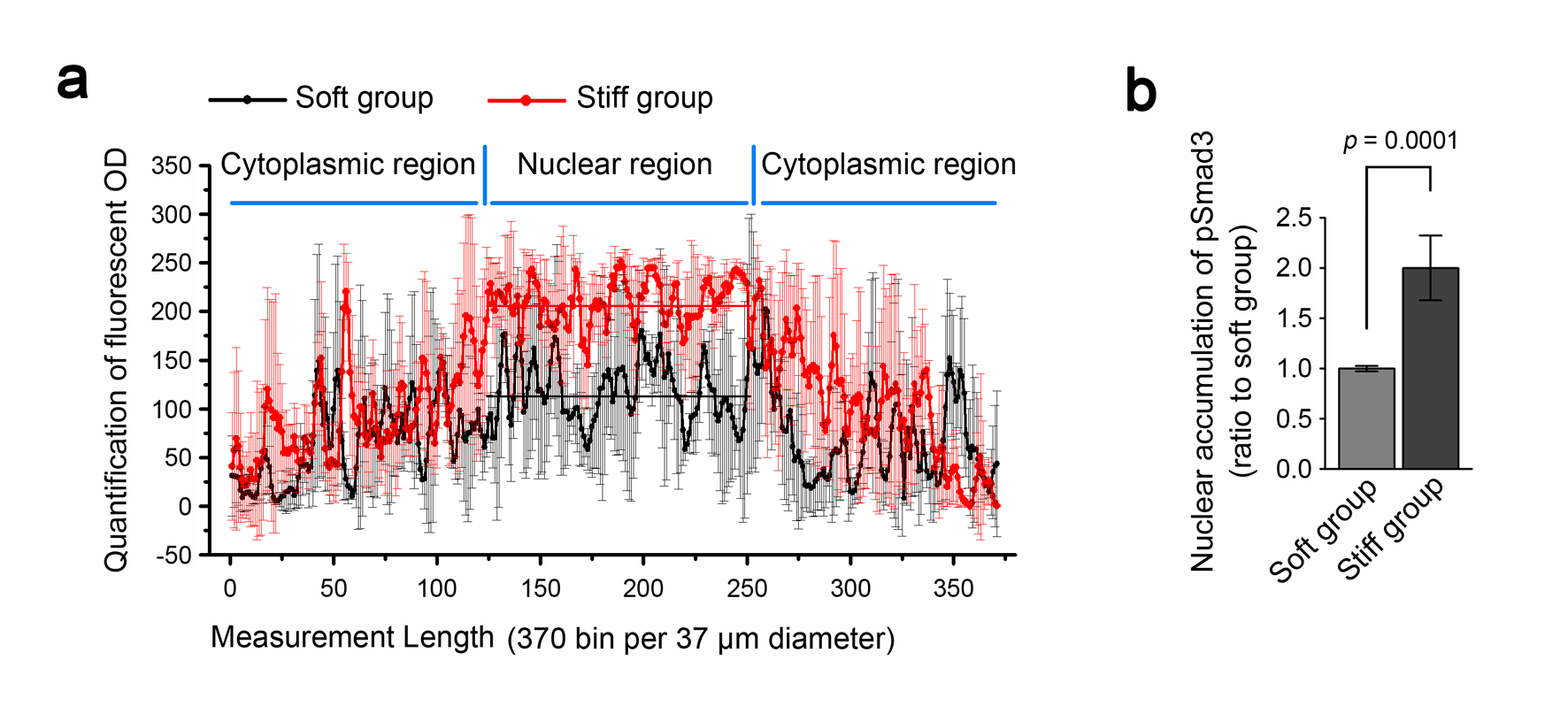


**Figure S19. Quantitative analysis of p-smad3 in chondrocytes in response to the soft/stiff substrates (This figure is related to Figure 6c).**

**a.** Linear fluorescent quantification indicating the nuclear accumulation of p-smad3 in chondrocytes in response to the soft/stiff substrates.

**b.** Quantification of total p-smad3 in chondrocytes in response to the substrates with different stiffnesses. Data were based on three independent experiments (n = 3).

**Figure S20**


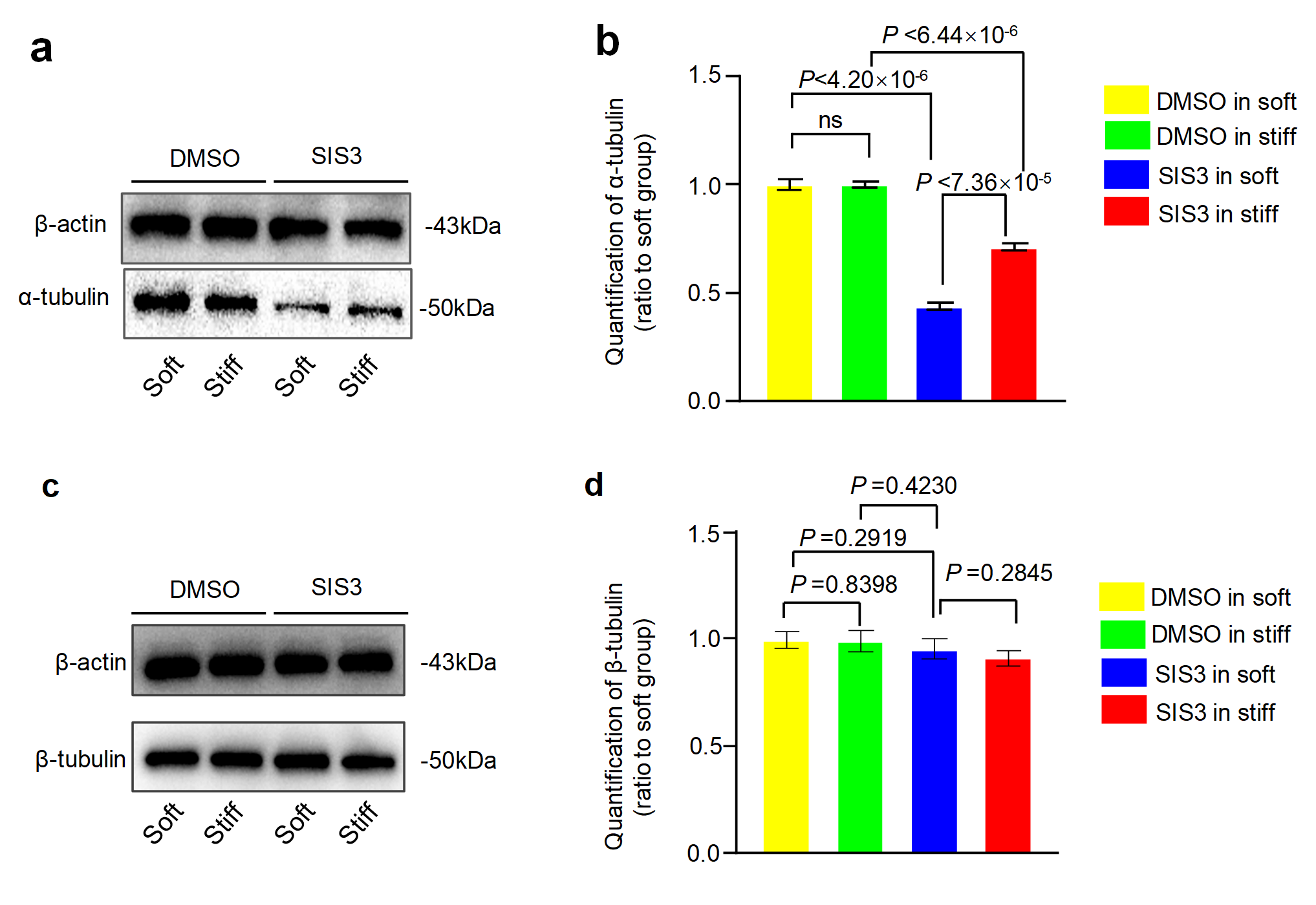


**Figure S20. The expression of α-tubulin but not β-tubulin in chondrcoytes seeded on the soft/stiff substrates induced by SIS3.**

**a.** Western blotting showing the expression change in α-tubulin in chondrcoytes seeded on the soft/stiff substrates induced by SIS3. The images are chosen from three independent experiments (n = 3).

**b.** Quantification of α-tubulin in chondrcoytes seeded on the soft/stiff substrates induced by SIS3. The quantifications of target proteins are calculated ratio to β-actin. Data are based on three independent experiments (n = 3).

**c.** Western blotting showing the expression change in β-tubulin in chondrcoytes seeded on the soft/stiff substrates induced by SIS3. The images are chosen from three independent experiments (n = 3).

**d.** Quantification of β-tubulin in chondrcoytes seeded on the soft/stiff substrates induced by SIS3. The quantifications of target proteins are calculated ratio to β-actin. Data are based on three independent experiments (n = 3).

**Figure S21**


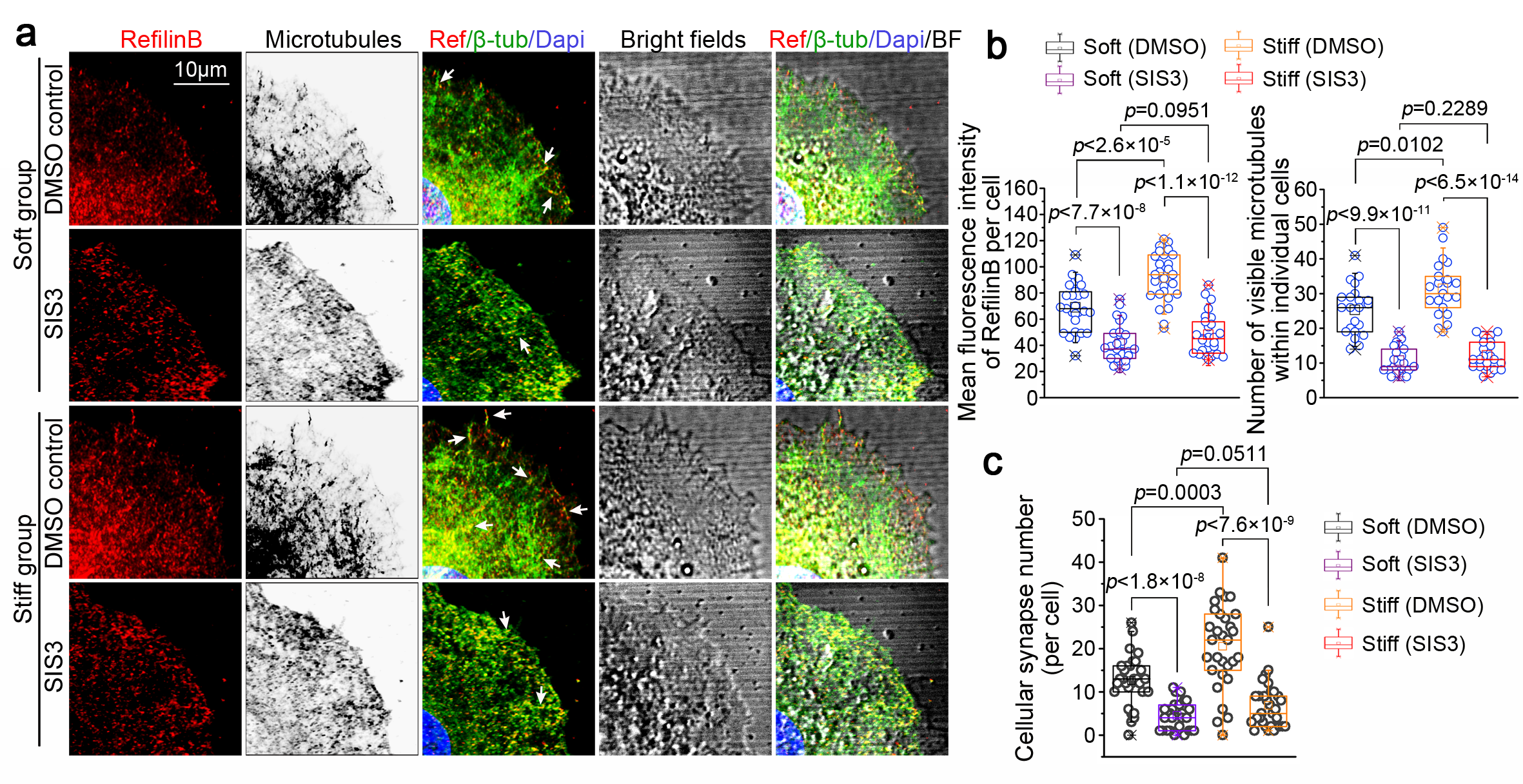


**Figure S21. The influence of SIS3 on refilinB protein, microtubules and cell spreading of chondrocytes seeded on the soft/stiff substrates.**

**a.** Representative CLSM images showing the changes of refilinB, microtubules and cell spreading of individual chondrocytes on soft/stiff substrates by SIS3 treatment (n = 4). White arrows indicate the microtubule morphology in the cytoplasm.

**b.** Quantitative analysis of refilinB (left) and microtubules (right) in chondrocytes on soft/stiff substrates by SIS3 treatment in (a). Mean fluorescence intensity of refilinB in chondrocytes is based on 27 cells from 4 independent experiments, and visible microtubule number is based on 22 cells from 4 independent experiments.

**c.** Box & whisker plot indicating cellular synapse changes in chondrocytes seeded on soft/stiff substrates after treatment with SIS3. The results are based on 30 cells from 4 independent experiments.

**Figure S22.**


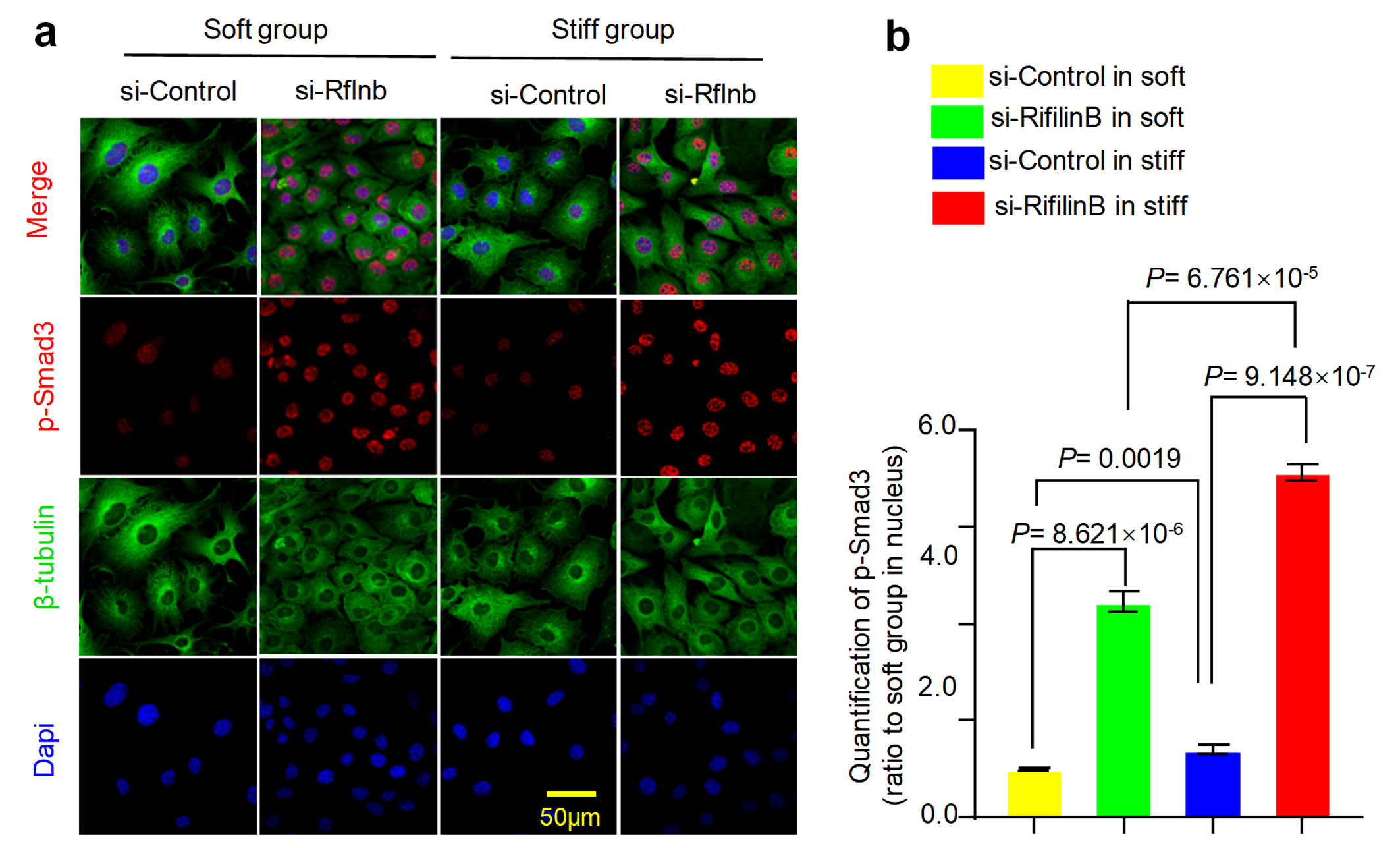


**Figure S22. The expression changes of p-smad3 in chondrocytes seeded on the soft/stiff substrates after refilinB silencing.**

**a.** Immunofluorescence showing the expression change in p-smad3 in chondrocytes after refilinB silencing. Images are chosen based on three independent experiments (n = 3).

**b.** Fluorescence quantification analysis confirming the change in p-smad3 in chondrocytes after refilinB silencing. Data are based on three independent experiments (n = 3). Significance data presented are based on two-tailed Student’s t tests.

**Figure S23**


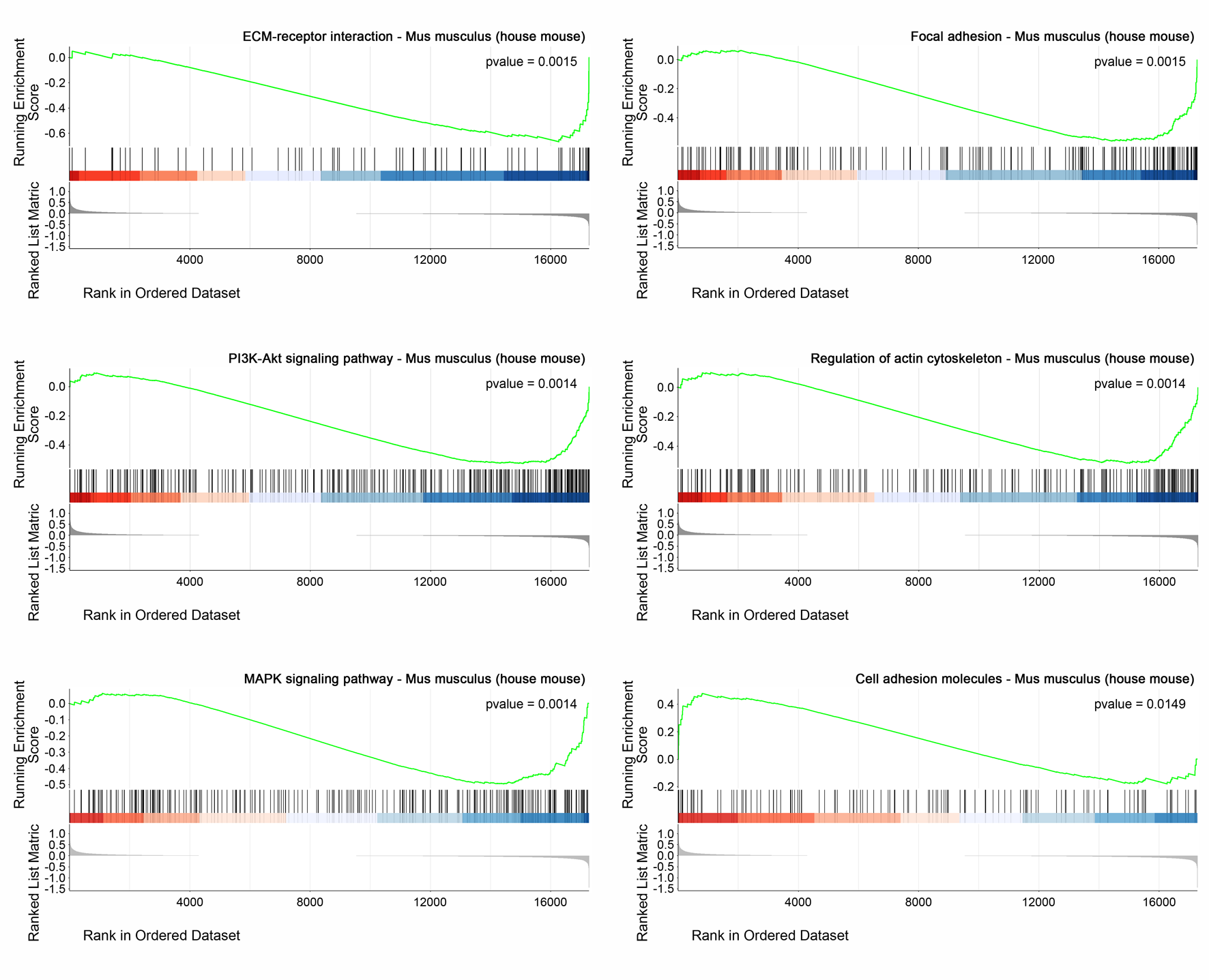


**Figure S23.** Enrichment plots from gene set enrichment analysis (GSEA) showing the signaling pathways, including ECM-receptor interaction, focal adhesion, PI3K-Akt signaling, regulation of actin cytoskeleton, MAPK signaling and cell adhesion molecules, involved in chondrocytes in response to substrates with different stiffnesses by single-cell RNA sequencing. The profile of gene candidates (source data) indicates the triggering of integrins and integrin-linked kinase (ILK) in chondrocytes in response to the soft/stiff substrates.

**Figure S24**


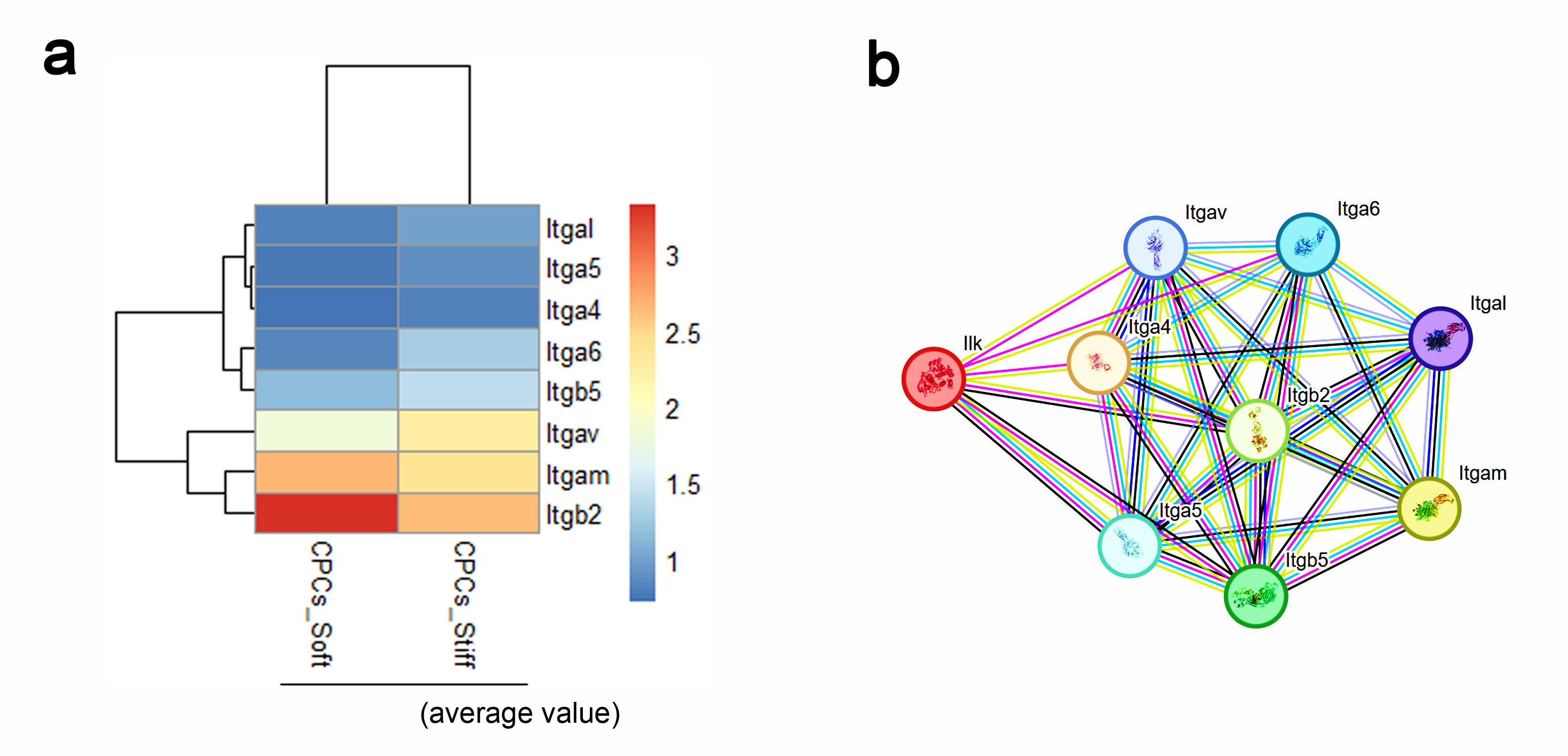


**Figure S24. Interaction between ILK and mechanosensative integrins in chondrocytes in response to substrate stiffness.**

**a.** Pheatmap showing the changes of integrins in chondrocytes in response to the soft/stiff substrates based on scRNA-seq. The data were presented as average values of expressions. These data changes were all based on p < 0.05.

**b.** Protein interaction showing the relationship between ILK and changed integrins in chondrocytes in response to substrate stiffness. The analysis of protein interaction was generated by using online STRING.

**Figure S25**


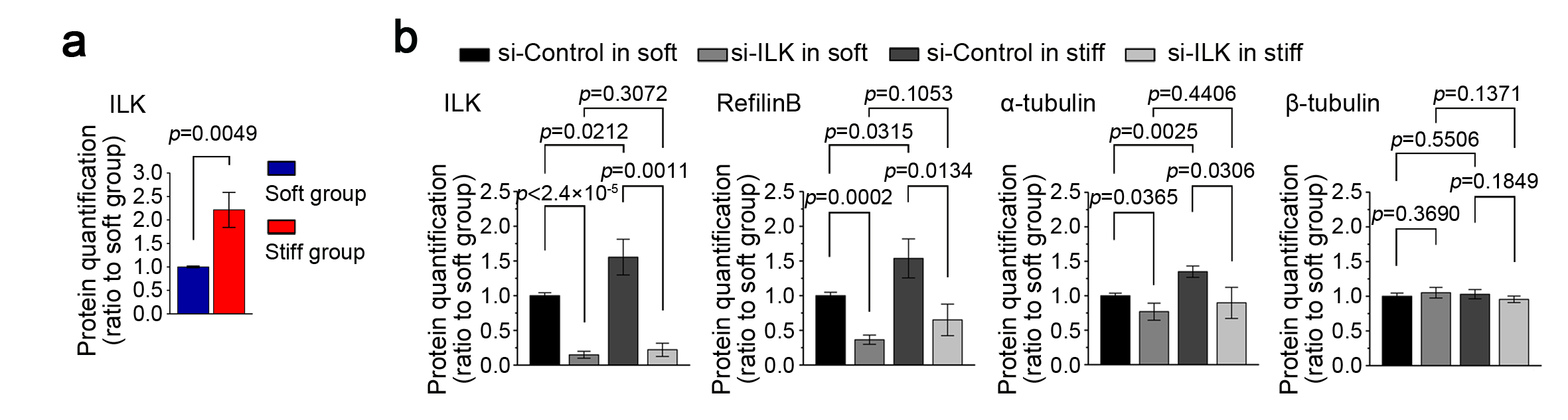


**Figure S25. Supplementary quantitative analysis of proteins in chondrocytes seeded onto the soft/stiff substrates induced by si-ILK.**

**a.** Quantitative analysis of ILK in chondrocytes in response to soft/stiff substrates. The data are based on three independent experiments (n = 3). The data are referred to Figure 7c.

**b.** Quantitative analysis of ILK, refilinB and tubulins in chondrocytes in response to soft/stiff substrates by si-ILK. The data are based on three independent experiments (n = 3). The data are referred to Figure 7d.

**Figure S26**


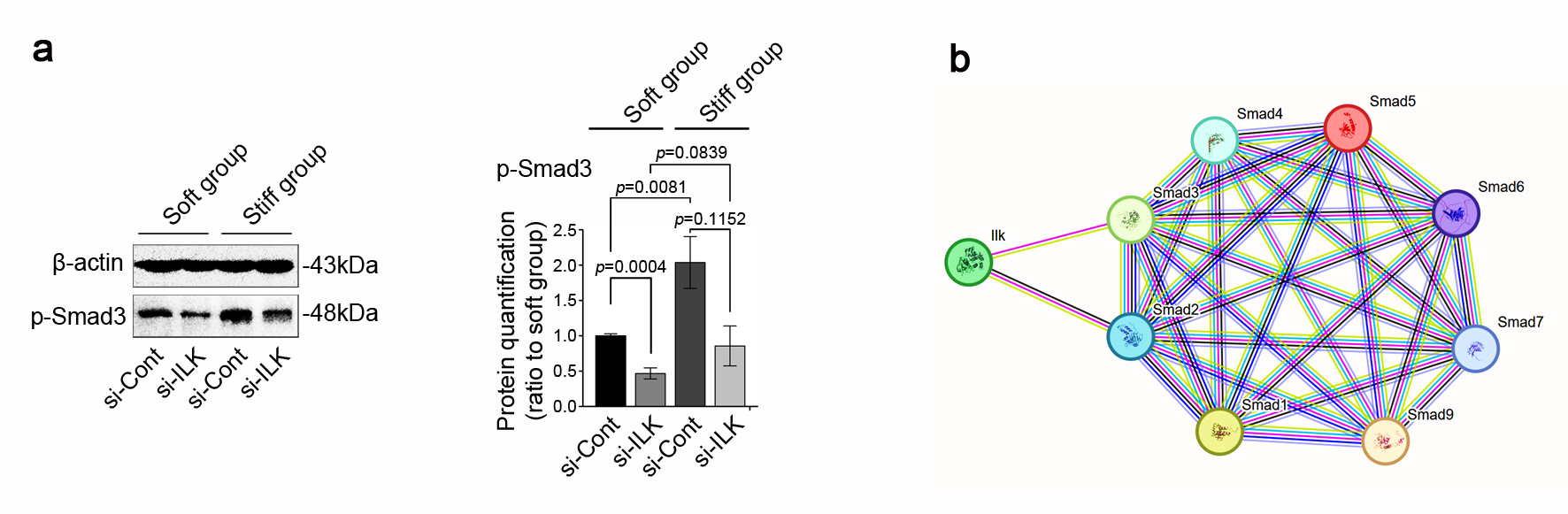


**Figure S26. The LIK knockdown by siRNA reduced the protein expression of p-Smad3 in chondrocytes on the soft/stiff substrates.**

**a.** Western blotting showing the protein changes of p-Smad3 in chondrocytes by si-ILK (left). Quantitative analysis showing ILK knockdown reduced the expression of p-Smad3 in chondrocytes by si-ILK (right). The quantifications of p-smad3 is calculated ratio to β-actin. Data were based on three independent experiments (n = 3). Significance data presented are based on two-tailed Student’s t tests.

**b.** Protein-protein interaction showing the correlation between ILK and smad signaling. The interaction was generated by online STRING based on changed genes in chondrocytes in response to substrate stiffness.
